# Pharmacological targeting of MTHFD2 suppresses acute myeloid leukemia by inducing thymidine depletion and replication stress

**DOI:** 10.1038/s43018-022-00331-y

**Published:** 2022-02-28

**Authors:** Nadilly Bonagas, Nina M. S. Gustafsson, Martin Henriksson, Petra Marttila, Robert Gustafsson, Elisée Wiita, Sanjay Borhade, Alanna C. Green, Karl S. A. Vallin, Antonio Sarno, Richard Svensson, Camilla Göktürk, Therese Pham, Ann-Sofie Jemth, Olga Loseva, Victoria Cookson, Nicole Kiweler, Lars Sandberg, Azita Rasti, Judith E. Unterlass, Martin Haraldsson, Yasmin Andersson, Emma R. Scaletti, Christoffer Bengtsson, Cynthia B. J. Paulin, Kumar Sanjiv, Eldar Abdurakhmanov, Linda Pudelko, Ben Kunz, Matthieu Desroses, Petar Iliev, Katarina Färnegårdh, Andreas Krämer, Neeraj Garg, Maurice Michel, Sara Häggblad, Malin Jarvius, Christina Kalderén, Amanda Bögedahl Jensen, Ingrid Almlöf, Stella Karsten, Si Min Zhang, Maria Häggblad, Anders Eriksson, Jianping Liu, Björn Glinghammar, Natalia Nekhotiaeva, Fredrik Klingegård, Tobias Koolmeister, Ulf Martens, Sabin Llona-Minguez, Ruth Moulson, Helena Nordström, Vendela Parrow, Leif Dahllund, Birger Sjöberg, Irene L. Vargas, Duy Duc Vo, Johan Wannberg, Stefan Knapp, Hans E. Krokan, Per I. Arvidsson, Martin Scobie, Johannes Meiser, Pål Stenmark, Ulrika Warpman Berglund, Evert J. Homan, Thomas Helleday

**Affiliations:** 1 Science for Life Laboratory, Department of Oncology–Pathology, Karolinska Institutet, Solna, Sweden; 2 Department of Biochemistry & Biophysics, Stockholm University, Stockholm, Sweden; 3 Weston Park Cancer Centre, Department of Oncology and Metabolism, The Medical School, University of Sheffield, Sheffield, UK; 4 Department of Cancer Research and Molecular Medicine, Norwegian University of Science and Technology, Trondheim, Norway; 5 Uppsala University Drug Optimization and Pharmaceutical Profiling Platform, Department of Pharmacy, Uppsala University, Uppsala, Sweden; 6 Cancer Metabolism Group, Department of Oncology, Luxembourg Institute of Health, Luxembourg, Luxembourg; 7 Drug Discovery and Development Platform, Science for Life Laboratory, Department of Organic Chemistry, Stockholm University, Solna, Sweden; 8 Drug Discovery and Development Platform, Science for Life Laboratory, Department of Medical Biochemistry and Biophysics, Karolinska Institutet, Solna, Sweden; 9 Drug Discovery and Development Platform, Science for Life Laboratory, School of Engineering Sciences in Chemistry, Biotechnology and Health, Royal Institute of Technology, Solna, Sweden; 10 Department of Experimental Medical Science, Lund University, Lund, Sweden; 11 Drug Discovery and Development Platform, Science for Life Laboratory, Department of Chemistry—BMC, Uppsala University, Uppsala, Sweden; 12 Institute of Pharmaceutical Chemistry, Goethe University, Frankfurt, Germany; 13 Department of Medicinal Chemistry, Science for Life Laboratory, Uppsala University, Uppsala, Sweden; 14 Biochemical and Cellular Screening Facility, Science for Life Laboratory, Department of Biochemistry and Biophysics, Stockholm University, Solna, Sweden; 15 Department of Medical Sciences, Division of Cancer Pharmacology and Computational Medicine, Uppsala University, Uppsala, Sweden; 16 Karolinska High Throughput Centre, Department of Biosciences and Nutrition, Karolinska Institutet, Huddinge, Sweden

**Keywords:** Targeted therapies, DNA synthesis, Cancer metabolism, Cancer, Drug discovery

## Abstract

The folate metabolism enzyme MTHFD2 (methylenetetrahydrofolate dehydrogenase/cyclohydrolase) is consistently overexpressed in cancer but its roles are not fully characterized, and current candidate inhibitors have limited potency for clinical development. In the present study, we demonstrate a role for MTHFD2 in DNA replication and genomic stability in cancer cells, and perform a drug screen to identify potent and selective nanomolar MTHFD2 inhibitors; protein cocrystal structures demonstrated binding to the active site of MTHFD2 and target engagement. MTHFD2 inhibitors reduced replication fork speed and induced replication stress followed by S-phase arrest and apoptosis of acute myeloid leukemia cells in vitro and in vivo, with a therapeutic window spanning four orders of magnitude compared with nontumorigenic cells. Mechanistically, MTHFD2 inhibitors prevented thymidine production leading to misincorporation of uracil into DNA and replication stress. Overall, these results demonstrate a functional link between MTHFD2-dependent cancer metabolism and replication stress that can be exploited therapeutically with this new class of inhibitors.

## Abstract (web summary)

Helleday and colleagues describe a nanomolar MTHFD2 inhibitor that causes replication stress and DNA damage accumulation in cancer cells via thymidine depletion, demonstrating a potential therapeutic strategy in AML tumors in vivo.

## Main

Oncogene-induced replication stress (RS) and activation of the DNA-damage response (DDR) are common features in the early stages of cancer development, ultimately leading to genomic instability, mutations and cancer progression^1–5^. It is also well established that anticancer treatments using traditional chemotherapy agents or more contemporary DDR inhibitors such as poly(ADP-ribose) polymerase (PARP) inhibitors often cause RS as a consequence of their mechanism of action to kill cancer cells^6^. Most chemotherapy agents cause DNA lesions and RS in both normal and cancer cells, resulting in dose-limiting treatments. PARP inhibitors cause RS and toxicity specifically in cancer cells mutated in homologous recombination genes (for example, *BRCA1* and *BRCA2*) using the concept of synthetic lethality^7,8^. This specific toxicity to cancer cells has allowed the continuous use of well-tolerated PARP inhibitors as maintenance therapy, dramatically improving progression-free survival after first-line treatment in high-grade serious ovarian cancer^9^.

In the present study, we explored whether synthetic lethality and cancer-specific toxicity could be obtained by focusing on cancer-specific metabolic changes rather than a specific cancer mutation.

Altered cellular metabolism is a hallmark of cancer and reprogramming of the one-carbon pathway is a major driver of cancer cell proliferation, providing building blocks for biosynthesis of nucleotides, methylation reactions and redox homeostasis^10–14^. Indeed, the one-carbon metabolism enzyme MTHFD2 stands out as the most consistently overexpressed metabolic enzyme across human tumors^15^. MTHFD2 enzymatic function supports rapid cell proliferation during early embryogenesis, after which it is replaced by the mitochondrial protein MTHFD2L in healthy mature tissue^16–18^. The widespread reactivation of MTHFD2 in tumors suggests an isoform switch from MTHFD2L to MTHFD2 during cancer transformation. Consistent with its expression profile, knockdown of MTHFD2 decreases proliferation of tumor-derived cell lines independent of tissue of origin and prolongs survival in human xenograft and mouse acute myeloid leukemia (AML) models^15,19^. In the present study, we perform a comprehensive study to validate MTHFD2 as an anticancer target and develop potent MTHFD2 inhibitors to be used as tools to explore the mechanism of action of MTHFD2 in cancer, as well as to demonstrate their therapeutic potential.

## Results

### MTHFD2 prevents replication stress in cancer cells

**Fig. 1:**
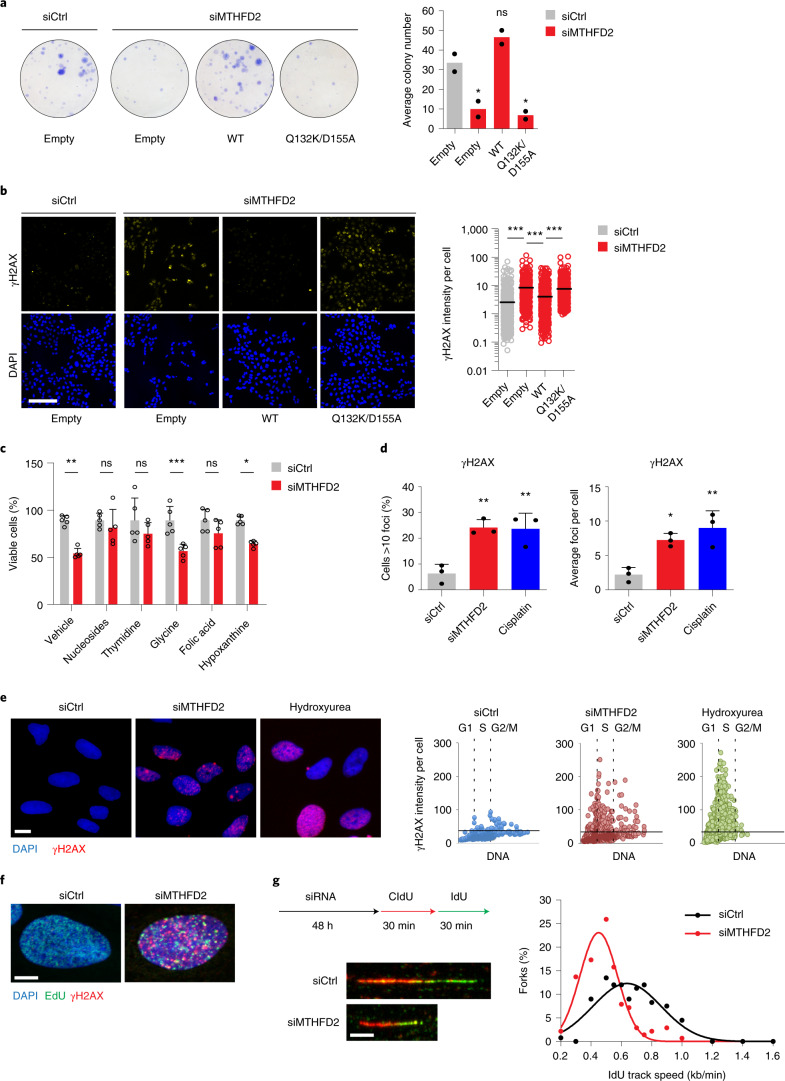
MTHFD2 supports cancer cell survival through DNA replication. **a**, Colony formation of U2OS cells on siMTHFD2 and coexpression of WT MTHFD2, catalytically dead mutant (Gln132Lys/Asp155Ala) or empty vector. Bars represent means (*n* = 2 independent experiments). **b**, Confocal analysis of DNA damage, γH2AX, on 24-h siMTHFD2 and coexpression of WT MTHFD2 or the Gln132Lys/Asp155Ala mutant in U2OS cells. Scale bar, 100 μm. Dot plots show γH2AX intensity per cell; bars represent means (*n* (cells) = 1,020 (siCtrl), 612 (siMTHFD2), 791 (WT) and 1,269 (Gln132Lys/Asp155Ala)). ^***^P < 0.001; one-way ANOVA (*F* = 141.9, degrees of freedom (d.f.) = 3). **c**, Metabolic rescue of siMTHFD2 U2OS cell viability with nucleosides (adenosine 30 μM, cytidine 30 μM, uridine 30 μM, guanosine 30 μM), thymidine 250 μM, glycine 250 μM, folic acid 250 μM or hypoxanthine 250 μM (72 h). Bars represent means + s.d. (*n* = 5 independent cell cultures). ^*^P = 0.0161, ^**^P = 0.0026, ^***^P < 0.001; two-way ANOVA (*F*_siRNA_ = 45.42, *F*_treatment_ = 7.466, d.f._siRNA_ = 1, d.f._treatment_ = 5); NS, not significant. One of two independent experiments is shown. **d**, Confocal analysis of γH2AX in U2OS cells after siMTHFD2 (24 h) or cisplatin treatment (10 μM, 16 h) (*n* = 3 independent experiments (>500 cells per treatment)); the bars represent means + s.d. Cells >10 foci: ^**^P_siMTHFD2_ = 0.0048, ^**^P_Cisplatin_ = 0.0055; one-way ANOVA (*F* = 15.69, d.f. = 2). Average foci per cell: ^*^P = 0.0172, ^**^P = 0.0043; one-way ANOVA (*F* = 13.64, d.f. = 2). **e**, Image-based cytometry analysis of γH2AX in U2OS cells on siMTHFD2 (24 h) or hydroxyurea (HU; 2 mM, 2 h). Scale bar, 50 μm. Single-cell analysis shows γH2AX intensity plotted against DNA content (DAPI) (*n* (cells) = 1,670 (siCtrl), 3,432 (siMTHFD2), 2,731 (HU)). **f**, Confocal analysis of DNA damage at replication sites, γH2AX and EdU, on 48 h siMTHFD2 in U2OS cells. Scale bar, 50 μm. **g**, DNA fiber assay on siMTHFD2 (48 h) in U2OS cells. Scale bar, 5 μm. Graphs show the distribution of RF speed per treatment (*n* (fibers/condition) = 133 (siCtrl) and 137 (siMTHFD2)). One of three independent experiments is shown. Source data

Although MTHFD2 RNA interference (RNAi) knockdown has been shown to block proliferation of cancer cells^15,19–21^, these experiments have not been complemented with rescue expression of RNAi-resistant MTHFD2. Furthermore, these studies did not address the requirement for enzymatic activity of MTHFD2, or how loss of MTHFD2 mechanistically contributes to cancer cell death. In the present study, we were able to rescue long-term survival of U2OS cancer cells on MTHFD2 small interfering (si)RNA treatment by reintroducing wild-type (WT) siRNA-resistant MTHFD2, but not the siRNA-resistant, catalytically dead construct (Gln132Lys/Asp155Ala) carrying point mutations in the tetrahyrofolate (THF)-binding pocket (Fig. 1a). Thus, the substrate (THF)-binding activity is key for MTHFD2-mediated cancer cell proliferation. We observed that MTHFD2 siRNA knockdown induced the DNA-damage marker Ser139-phosphorylated histone H2AX (γH2AX), which was rescued by expression of siRNA-resistant WT MTHFD2, but not Gln132Lys/Asp155Ala (Fig. 1b), further supporting the importance of the THF-binding activity of MTHFD2.

**Extended Data Fig. 1:**
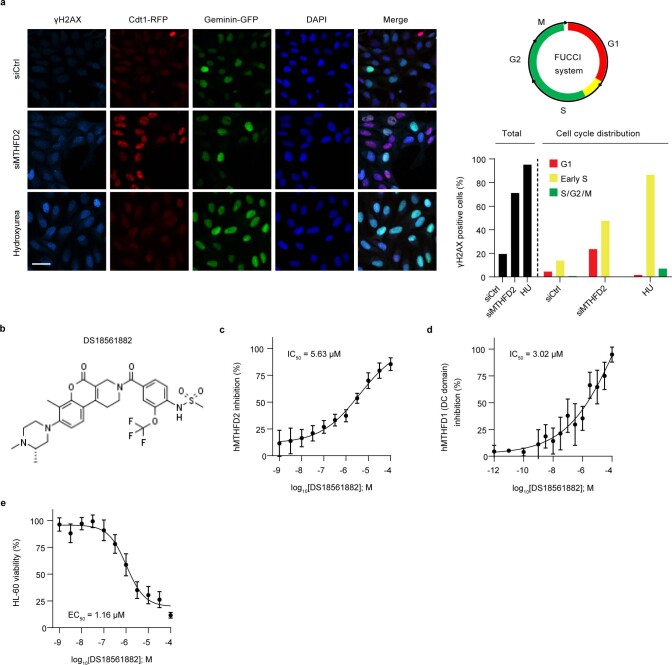
MTHFD2 inhibitor DS18561882 is a weak and unselective inhibitor of MTHFD2. (a) Depletion of MTHFD2 induces accumulation of DNA damage in early S phase. Confocal analysis of DNA damage (γH2AX) in U2OS cells expressing the FUCCI sensor system following siRNA knock-down of MTHFD2 (24 h) or hydroxyurea (HU) treatment (2 mM, 24 h). Scale bar, 50 μm. Shown is a representative out of three independent experiments; n (cells/treatment) = 335 (siCtrl), 229 (siMTHFD2), 186 (HU). (b) Structure of the small molecule DS18561882. (c) Dose-response curve (DRC) for the biochemical inhibition of hMTHFD2 by DS18561882 assessed using the NAD(P)H-Glo assay. Graph represents pooled results from four independent experiments carried out in duplicate, with average IC_50_ value reported (n = 4). Data are displayed as means ± SD. (d) DRC for the biochemical inhibition of hMTHFD1 (DC domain) by DS18561882 assessed using the NAD(P)H-Glo assay. Graph represents pooled results from four independent experiments carried out in duplicate, with average IC50 value reported (n = 4). Data are displayed as means ± SD. (e) Cell viability DRC for DS18561882 in HL-60 cells, evaluated after 96 h. Graph represents pooled results from four independent experiments carried out in duplicate, with average EC_50_ value reported (n = 4). Data are displayed as means ± SD. Source data

As MTHFD2 generates metabolites to sustain biomass production in cancer, we supplemented metabolites to MTHFD2 knockdown cells to identify potential rescue. Both nucleosides and folic acid rescued viability, but not glycine or hypoxanthine (Fig. 1c). Importantly, addition of thymidine alone rescued viability to MTHFD2 loss, suggesting that the antiproliferative effects may be related to thymine-less death. This prompted us to assess RS-induced DNA damage by analyzing γH2AX levels. We found that γH2AX foci were induced on siMTHFD2 (Fig. 1d), which were predominantly accumulated during the S phase of the cell cycle after MTHFD2 knockdown, indicative of RS (Fig. 1e). Using the FUCCI (fluorescent ubiquitination-based cell cycle indicator) cell-cycle reporter system, we were able to confirm the early S-phase-specific accumulation of DNA damage on MTHFD2 siRNA treatment (Extended Data Fig. 1). Furthermore, we observed γH2AX foci specifically at nascent replication forks by colocalization with newly incorporated 5-ethynyl-2′-deoxyuridine (EdU) foci on MTHFD2 knockdown (Fig. 1f), suggesting that the DNA damage occurred at replication forks (RFs). To further evaluate potential replication impairments on MTHFD2 silencing, we used the DNA fiber assay and detected shorter fibers as a result of decreased fork speed on siMTHFD2 treatment compared with siControl (Fig. 1g). Taken together, these results show that MTHFD2 prevents RS in cancer cells.

### Development of first-in-class MTHFD2 inhibitors

We previously solved the crystal structure of human MTHFD2 in a complex with a weak inhibitor, LY345899 (ref. ^22^). Although other weak inhibitors of MTHFD2 have recently been published (for example, DS18561882; Extended Data Fig. 1)^23,24^, a potent inhibitor to validate MTHFD2 as an anticancer target in cells or in vivo still remains to be described.

**Extended Data Fig. 2:**
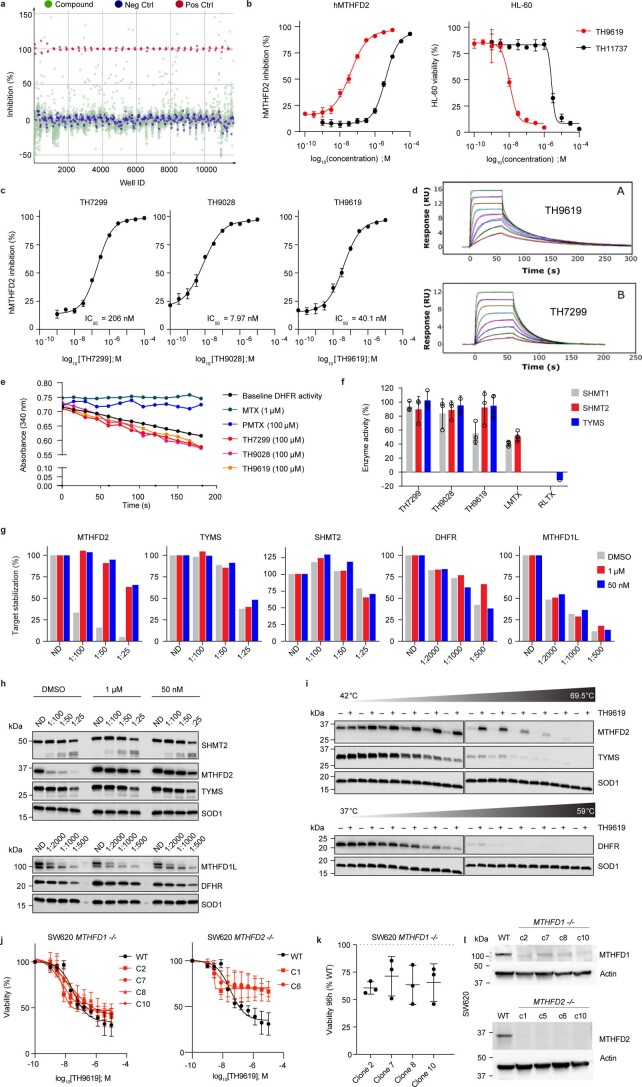
Biochemical characterization of MTHFD2i target selectivity. (a) HTS of ~11,000 compounds (10 µM). LY345899 served as positive control (red) and assay buffer as negative control (blue). (b) Representative inhibition DRCs for human recombinant MTHFD2 (hMTHFD2) and HL-60 cell viability upon TH9619 and TH11737, (S)- and (R)- enantiomers respectively. Data is displayed as means ± SD, n = 6 (TH11737) and n = 2 (TH9619). (c) Representative inhibition DRCs for hMTHFD2 by TH7299, TH9028 and TH9619. IC_50_ values correspond to the representative run (n = 2). Data are displayed as means ± SD. (d) SPR analysis of MTHFD2i and hMTHFD2. Black lines correspond to a fitted 1:1 Langmuir interaction model. RU: response units, s: seconds. Shown is a representative out of two independent experiments. (e) DHFR activity upon MTHFD2i (100 µM) compared to DHFR inhibitors methotrexate (MTX) and pemetrexed (PMTX). Data is displayed as means, n = 2. (f) SHMT1, SHMT2 and TYMS activity upon MTHFD2i (100 μM) compared to lometrexol (LMTX) and raltitrexed (RLTX). Bars represent means ± SD. From left to right, n (replicates/condition) = 3; 3; 3; 3; 3; 2; 3; 3; 4; 4; 4; 1. (g) DARTS analysis upon TH9619 treatment of HL-60 cells. Protein levels were normalized to non-digested (ND) controls. Representative graphs are shown for one out of two independent experiments. (h) Representative immunoblot images corresponding to DARTS experiment in (g) are shown for one out of two independent experiments. (i) CETSA of MTHFD2, TYMS and DHFR upon TH9619 treatment (1 µM). Protein levels were normalized against SOD1. Shown is a representative image out of two independent experiments. (j) Viability of MTHFD1-/- and MTHFD2-/- knockout SW620 cell clones or wildtype cells treated with TH9619 for 96 h. Data from three independent experiments are displayed as means ± SD, n = 3. (k) Viability of MTHFD1-/- clones at 96 h relative to wildtype SW620 cells. Data from three independent experiments are displayed as means ± SD, n = 3. (l) Representative images of MTHFD1 and MTHFD2 expression in wildtype SW620 cells compared to MTHFD1-/- and MTHFD2-/- knockout clones, respectively. Representative graphs are shown for one out of three independent experiments. Source data

In the present study, we set up a biochemical assay^22^ that quantifies the amount of NADH produced from NAD^+^ by MTHFD2 on oxidation of the substrate folitixorin and screened over 500,000 lead-like compounds in 3 screening campaigns, including 443,000 compounds from the European Lead Factory (Supplementary Table 1). The few hits observed in the screens (Extended Data Fig. 2) also quenched the assay detection signal in the absence of MTHFD2, or contained promiscuous scaffolds or functional groups suggesting nonspecific assay interference^25^. Hence, none of the hits was deemed suitable for hit expansion.

**Fig. 2:**
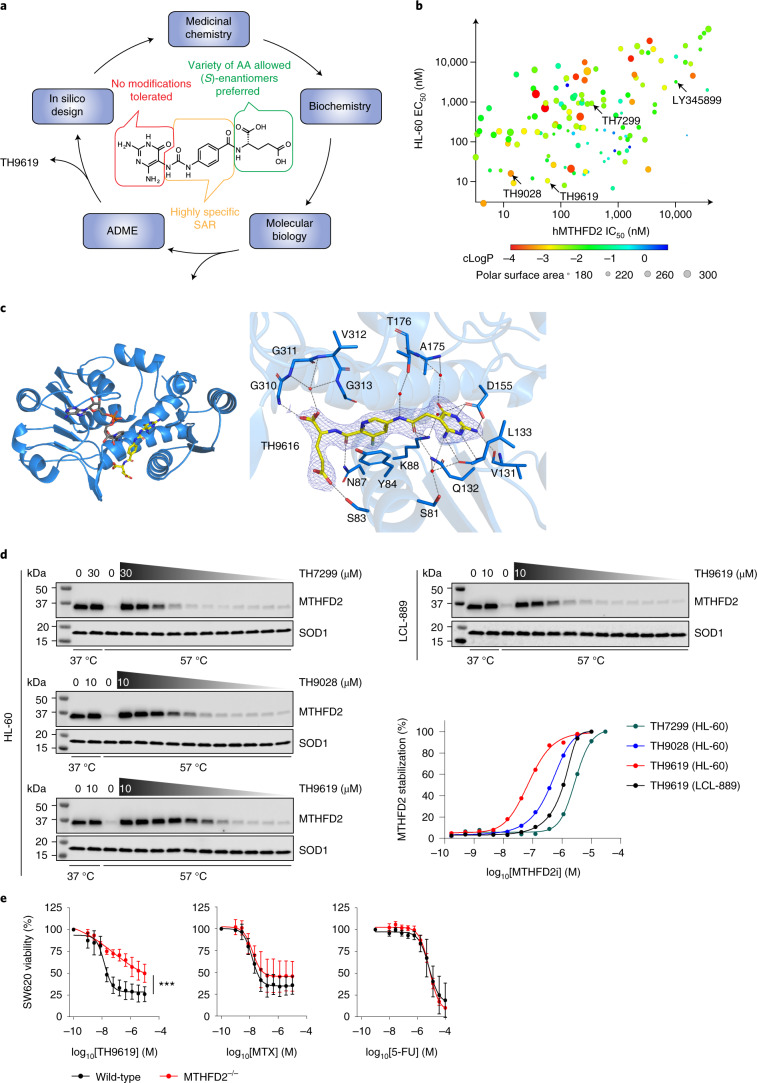
Development of first-in-class MTHFD2 inhibitors. **a**, Schematic description of the drug discovery process toward the identification of MTHFD2 inhibitors. Intensive SAR-guided lead optimization efforts resulted in a series of low-nanomolar, cell-active MTHFD2 inhibitors, exemplified in the present study by TH9028 and TH9619. AA, amino acid. **b**, Relationship between enzymatic IC_50_ and HL-60 EC_50_ of MTHFD2 inhibitors. In total, >300 compounds were synthesized, biochemically characterized and evaluated for inhibition of viability in cells. **c**, Cocrystal structures of TH7299 and TH9619 bound to human MTHFD2. To the left, the overall structure of MTHFD2 shown as a blue cartoon representation. The bound TH7299 inhibitor and NAD^+^ cofactor are shown as sticks, colored yellow and gray, respectively. To the right are structural details of TH9619 (yellow) binding to MTHFD2. Important residues in the binding site are marked. Water molecules are displayed as red spheres, and hydrogen bond interactions are shown as dashed lines. The 2*F*_o_–*F*_c_ electron density map around TH9619 is contoured at 1.3 σ in blue and the *F*_o_–*F*_c_ maps are contoured at −3.0 σ in red and +3.0 σ in green (Supplementary Table 3). Figures were produced using PyMOL. **d**, Isothermal dose–response fingerprint CETSA_ITDRF_ showing target engagement and thermal stabilization of MTHFD2 by TH7299, TH9028 and TH9619 in intact HL-60 and LCL-889 cells. [MTHFD2i], concentration of MTHFD2 inhibitor. The graph shows the nondenatured MTHFD2 fraction at 57 °C, displayed as means (*n* = 2). Representative images are shown for one of two independent experiments. **e**, Viability dose–response curves of WT and CRISPR–Cas9 *MTHFD2*^−/−^ knockout SW620 colorectal cancer cells on treatment with MTHFD2 inhibitor TH9619, MTX or 5-FU, evaluated after 96 h. Data are pooled from three independent experiments, represented as means ± s.d. One-way, extra sum-of-squares *F* test: TH9619 (^***^P < 0.001, *F* = 40.65, d.f. = 1), MTX (*P* = 0.0448, *F* = 2.607, d.f. = 1) and 5-FU (*P* = 0.1499, *F* = 1.757, d.f. = 1). Source data

**Extended Data Fig. 3:**
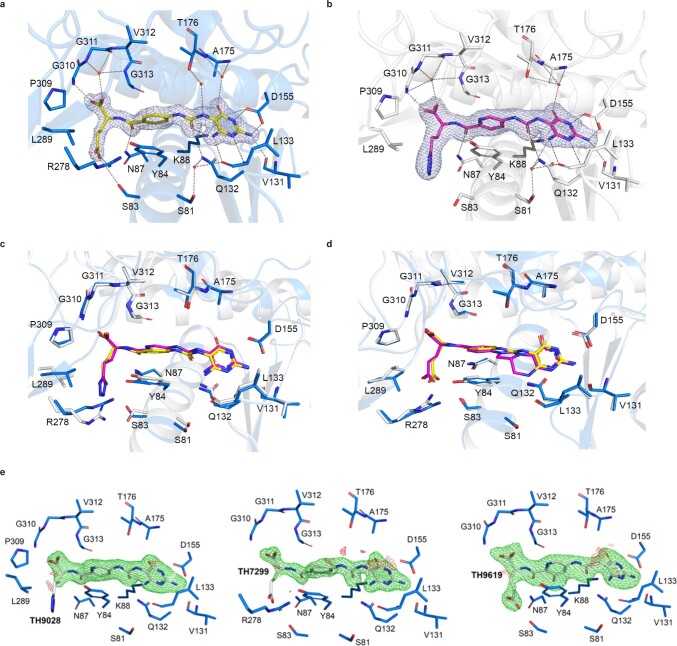
Co-crystal structures of TH7299 and TH9028 bound to human MTHFD2. (a, b) Structural details of (a) TH7299 (yellow) and (b) TH9028 (magenta) binding to MTHFD2. Important residues in the binding site are marked. Water molecules are displayed as red spheres. Hydrogen bond interactions are shown as dashed lines. The 2*F*_o_*-F*_c_ electron density maps around TH7299 and TH9028 are contoured at 1.3σ (blue) and the *F*_o_*-F*_c_ maps are contoured at −3.0σ (red) and +3.0σ (green). (c) Comparison of the binding sites of MTHFD2 bound with TH7299 (blue) and TH9028 (white). TH7299 and TH9028 are shown as sticks colored yellow and magenta, respectively. (d) Comparison of the binding sites of MTHFD2 bound with TH7299 (blue) and LY345899 (white). TH7299 and LY345899 are shown as sticks colored yellow and magenta, respectively. Figures were produced with PyMOL. (e) *F*_o_*-F*_c_ electron density maps prior to ligand refinement for TH9028, TH7299 and TH9619. Important residues in the binding site pocket are marked. The *F*_o_*-F*_c_ maps are contoured at −3.0σ (red) and +3.0σ (green). Figures were produced with PyMOL.

As an alternative approach, we adopted a substrate-guided lead optimization strategy to find suitable small molecules with the ability to potently inhibit MTHFD2 activity (Fig. 2a). Simplification of the tricyclic core of LY345899 led to TH7299, earlier described as the bacterial FolD inhibitor 9L9 (ref. ^26^), with improved activity toward MTHFD2. Systematic exploration of the structure–activity relationships (SARs) by iterative cycles of structure-guided design, synthesis and evaluation of biochemical and cellular activity revealed that: (1) the diaminopyrimidine head group did not tolerate any structural modifications, (2) the urea linker and central phenyl ring displayed highly specific SARs and (3) the glutamate tail could be replaced by a variety of amino acids and isosteres. A strong preference for the natural (*S*) configuration of the glutamate chiral center was observed with respect to both MTHFD2 inhibition and cellular activity (Extended Data Fig. 2). These efforts resulted in the identification of a series of compounds displaying low-nanomolar affinity toward MTHFD2 in enzymatic and binding assays, as well as increased cell activity compared with LY345899 and TH7299 (Table 1, Extended Data Fig. 2 and Supplementary Table 2). Despite the hydrophilic nature of the compounds, as reflected by their low cLog*P* (calculated logarithm of partition coefficient) and large polar surface areas, a fair correlation between biochemical and cellular activity was found (Fig. 2b), suggesting that active transport is involved in their cellular activity. The tool compound TH7299, as well as two of the most promising candidates, TH9028 and TH9619, were chosen to investigate the MTHFD2 mechanism of action and cancer biology. Furthermore, we determined the cocrystal structures of TH7299, TH9028 and TH9619 in complexes with human MTHFD2 to assess their binding mode and identify critical interactions in the binding pocket (Fig. 2c, Extended Data Fig. 3 and Supplementary Table 3).

**Table 1:** Chemical structures of MTHFD2 inhibitors

	Structure	IC_50_ (nM)	EC_50_ (nM)
LY345899		6396	4005
TH7299		254	1110
TH9028		11	17
TH9619		47	11

Chemical structures of MTHFD2 inhibitors LY345899, TH7299, TH9028 and TH9619. Inhibition of MTHFD2 enzymatic activity in biochemical assays and efficacy on HL-60 cell viability are listed as IC_50_ and EC_50_ values, respectively.

We observed that the compounds bound to the MTHFD2 target in HL-60 cells, using drug affinity responsive target stability (DARTS) and cellular thermal shift assays (CETSAs), determine target stabilization against proteolytic and thermal protein degradation, respectively (Fig. 2d and Extended Data Fig. 2). Given the THF-like structure of our compounds, potential binding to other THF-dependent enzymes cannot be ruled out. In the present study, we show that, in contrast to the characteristic promiscuity and polypharmacology of antifolates such as methotrexate (MTX) and pemetrexed (PMTX)^27^, TH9619 displayed high selectivity toward binding and stabilizing MTHFD2 over other common folate metabolism targets such as dihydrofolate reductase (DHFR), thymidylate synthase (TYMS), serine hydroxymethyltransferase (SHMT) 1 and SHMT2 (Extended Data Fig. 2). Similar results were observed with TH7299 and TH9028 in biochemical and target engagement assays (Extended Data Fig. 2). Due to the sequence and structural similarity of MTHFD2, MTHFD2L and the dehydrogenase/cyclohydrolase (DC) domain of MTHFD1, these enzymes were also found to be targeted by these compounds in biochemical activity assays (Table 2).

**Table 2:** Biochemical inhibition of related MTHFD proteins

	MTHFD2 IC_50_ (nM)	MTHFD2L IC_50_ (nM)	MTHFD1-(DC) IC_50_ (nM)	D2/D1
LY345899	6,396	3,761	535	12
TH7299	254	126	89	3
TH9028	11	27	0.5	21
TH9619	47	47	16	3

Average biochemical IC_50_ values for MTHFD2 inhibitors tested on MTHFD2, MTHFD2L and MTHFD1-(DC) (*n* ≥ 3).

We could confirm MTHFD2 stabilization against thermal degradation on incubation with TH9619 in cell lysates and live cells using CETSA (Fig. 2d and Extended Data Fig. 2), demonstrating intracellular target engagement. At a predetermined temperature of 57 °C, isothermal dose–response fingerprint (CETSA_ITDRF_) analysis of TH7299, TH9028 and TH9619 in HL-60 cells accurately reflected their relative potencies in biochemical and viability assays, as well as demonstrating the difference in target stabilization potential for TH9619 in nontumorigenic lymphoblastoid cell line (LCL)-889 cells compared with HL-60 AML cells (Fig. 2d). Due to the high instability of the MTHFD2L and MTHFD1 proteins, establishing target engagement or surface plasmon resonance-binding assays for these proteins was not possible.

To evaluate whether targeting MTHFD2 was responsible for the observed toxicity on MTHFD2 inhibitors in cancer cells, we generated *MTHFD2*^−/−^ clustered regularly interspaced short palindromic repeats (CRISPR)–Cas9 knockout cells and found that they were several orders of magnitude more resistant to TH9619 than parental *MTHFD2* WT cells (Fig. 2e), but not *MTHFD1*^−/−^ cells (Extended Data Fig. 2). In contrast, response to MTX or 5-fluorouracil (5-FU) remained unchanged in *MTHFD2*^−/−^ cells. Furthermore, we generated and characterized individual CRISPR–Cas9 knockout clones of MTHFD1 and MTHFD2 and confirmed the resistance to TH9619 only in *MTHFD2*^−/−^ clones (Extended Data Fig. 2). Altogether, these data provide evidence that the compound series presented in the present study can be described as MTHFD2 inhibitors with low-nanomolar cellular potency, offering potential for use in anticancer therapy.

### MTHFD2 inhibitors display high potency and cancer selectivity

**Fig. 3:**
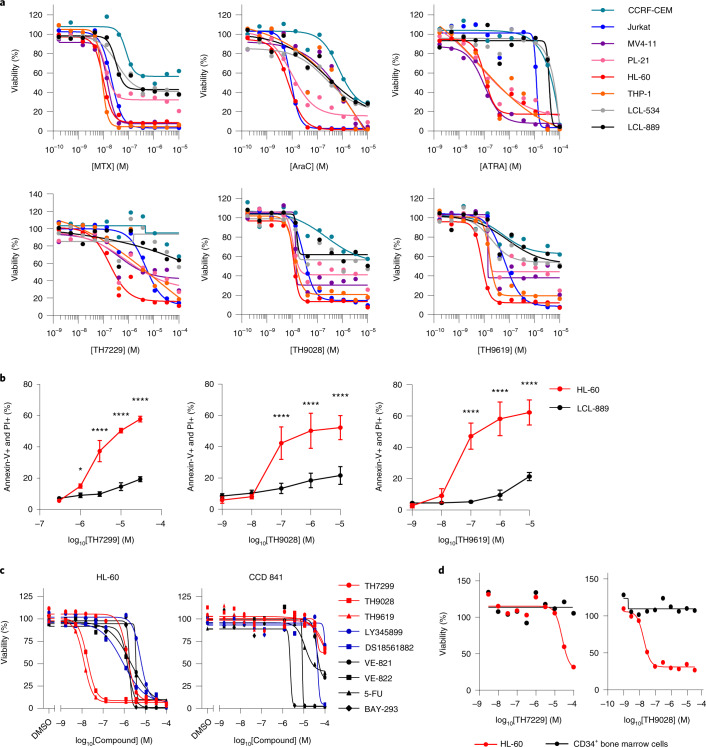
MTHFD2 inhibitors display high potency and cancer selectivity in AML models. **a**, Evaluation of the MTHFD2 inhibitors TH7299, TH9028 and TH9619 compared with MTX, AraC and ATRA on cell viability at 96 h of treatment across a panel of leukemia cell lines. LCLs LCL-534 and LCL-889 established from healthy primary B cells are shown as nontumorigenic controls (Extended Data Fig. 4). Data are shown as means (*n* = 2 independent cell cultures). A representative of two independent experiments is shown. **b**, Annexin-V/PI flow cytometry analysis of apoptosis in HL-60 and LCL-889 cells on TH7299, TH9028 or TH9619 for 96 h (Extended Data Fig. 4). Approximately 20,000 events were analyzed per condition. The quantification of double-positive (annexin-V positive and PI positive) late apoptotic populations is shown as pooled means ± s.d. from two independent experiments performed in duplicate. ^*^P = 0.0168, ^****^P < 0.0001; two-way ANOVA (*F*_TH7299_ = 650.9, *F*_TH9028_ = 80.27, *F*_TH9619_ = 246, d.f. = 1). **c**, Viability dose–response curves of CCD 841 normal colon epithelial cells compared with HL-60 cells on treatment with MTHFD2 inhibitors (TH7299, TH9028, TH9619, LY345899 and DS18561882), ATR inhibitors (VE-821, VE-822), 5-FU or KRASi (BAY-293) evaluated after 96 h (Extended Data Fig. 4). Data are shown as means (*n* = 2). A representative of two independent experiments is shown. **d**, Cell viability dose–response curves of primary CD34^+^ bone marrow cells from healthy donors compared with HL-60 cells on treatment with TH7299, TH9028 or DMSO, evaluated after 72 h. Data from one representative experiment are displayed as means (*n* = 2 independent cell cultures). Source data

**Extended Data Fig. 4:**
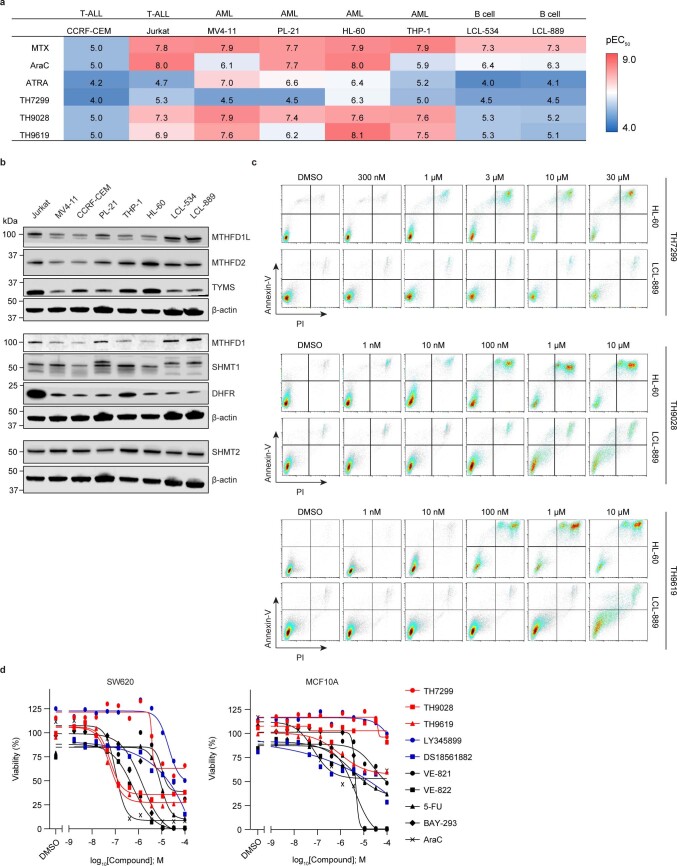
MTHFD2i display high cancer selectivity in AML models. (a) Table summarizing average pEC_50_ values from cell viability experiments upon treatment with MTHFD2 inhibitors TH7299, TH9028 and TH9619 compared to methotrexate (MTX), cytarabine (AraC) and all-*trans* retinoic acid (ATRA) across a panel of leukemia cell lines (n = 2). Lymphoblastic cell lines LCL-534 and LCL-889 established from healthy primary B cells are shown as non-malignant controls. (b) Basal protein levels of folate enzymes in AML cells compared to non-malignant LCL cells. β-actin was used as loading control. Shown is a representative out of two independent experiments. (c) Apoptosis as assessed by Annexin-V/PI staining of HL-60 and LCL-889 cells upon increasing doses of TH7299, TH9028 and TH9619 for 96 h. Approximately 20,000 events were gated per condition. Shown is a representative from two independent experiments performed in duplicate. (d) Cell viability dose-response curves of MCF10A non-transformed breast epithelial cells compared to SW620 colorectal cancer cells upon treatment with MTHFD2i (TH7299, TH9028, TH9619, LY345899, DS18561882), ATRi (VE-821, VE-822), 5-fluorouracil (5-FU), KRASi (BAY-293) or cytarabine (AraC), evaluated after 96 h. Data are shown as means, n = 2. Shown is a representative out of two independent experiments. Source data

As MTHFD2 has been validated with small hairpin RNA as a target in AML^19^, we next validated our inhibitors in a panel of leukemia cell lines, including acute lymphoblastic leukemia (ALL) and AML cells, as well as nontumorigenic LCLs established from healthy donors, LCL-534 and LCL-889. Recapitulating the situation in patients, the differentiation agent all-*trans*-retinoic acid (ATRA) was effective only in a subset of AML cell lines, with almost no effect on other leukemia types. As in the clinic, MTX and cytarabine (AraC) were highly potent and induced cancer cell death in several leukemia types; however, they also considerably affected the viability of nontumorigenic LCL cells. In contrast, TH9028 and TH9619 showed overall strong antiproliferative efficacy in AML cells and T-ALL Jurkat cells comparable to standard-of-care compounds, with reduced effect on LCL viability (Fig. 3a and Extended Data Fig. 4). Responses to MTHFD2 inhibitors also corresponded to MTHFD2 protein levels in AML cell lines compared with less sensitive LCL cells, whereas the expression profile of MTHFD1, SHMT1 or SHMT2 in these cells did not predict MTHFD2 inhibitor sensitivity (Extended Data Fig. 4).

Using annexin-V/PI staining, we confirmed that apoptosis was induced by MTHFD2 inhibitors in AML cells, but not LCL cells (Fig. 3b and Extended Data Fig. 4). Furthermore, nontransformed MCF10A breast epithelial cells and CCD 841 colonic epithelial cells were considerably less affected by MTHFD2 inhibitors at therapeutic doses, whereas reference compounds, including DS18561882 (ref. ^24^), impaired normal and cancer cell viability to similar extents (Fig. 3c and Extended Data Fig. 4). In line with these results, viability assays in hematopoietic stem-cell-enriched CD34^+^ bone marrow cells from healthy donors versus HL-60 cells revealed selective, dose-dependent inhibition of proliferation of cancer cells over nontransformed cells on MTHFD2 inhibitor treatment, demonstrating a therapeutic window for TH9028 spanning more than four orders of magnitude (Fig. 3d). Taken together, these data show enhanced selectivity of MTHFD2 inhibitors toward MTHFD2-expressing cancer cells with a wide therapeutic index.

### MTHFD2 inhibitors induce thymine-less DNA damage and apoptosis

**Fig. 4:**
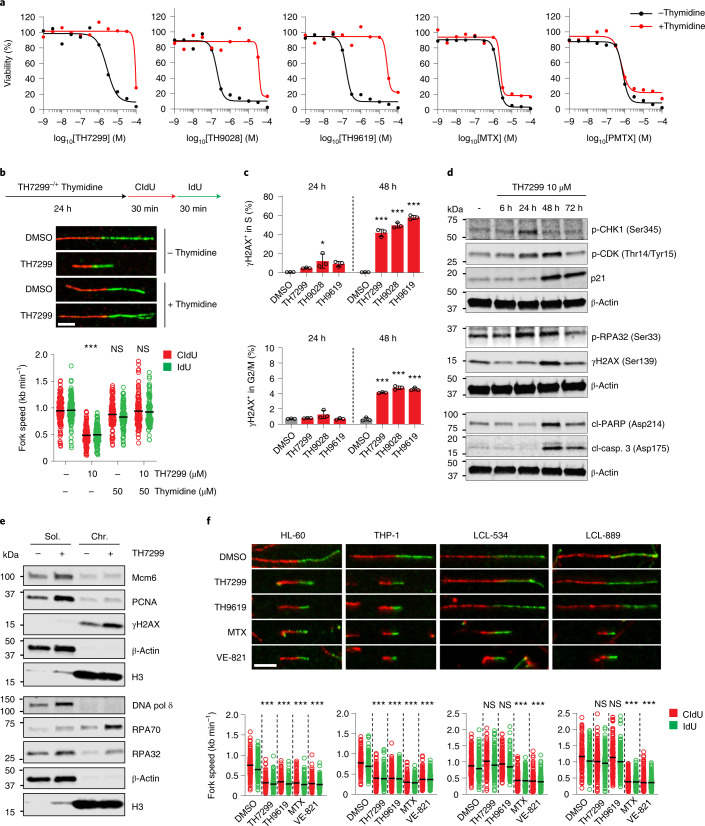
MTHFD2 inhibitors induce DNA damage and apoptosis via thymine-less death. **a**, HL-60 cell viability on MTHFD2 inhibitors, MTX or PMTX ± thymidine 50 μM at 96 h. Data from one representative experiment of two performed in duplicate are displayed as means. **b**, Thymidine rescue of DNA replication on TH7299 in THP-1 cells. Scale bar, 5 μm. Scatter dot plots show the mean and distribution of RF speeds per treatment (*n* (fibers per condition) = 105 (except TH7299 + thymidine, *n* = 100)). ^***^P < 0.001; one-way ANOVA (*F* = 99.79, d.f. = 3). NS, not significant. **c**, Cell-cycle analysis of DNA damage (γH2AX) on MTHFD2 inhibitors (TH7299 3 μM, TH9028/TH9619 50 nM) in HL-60 cells. Per sample, at least 15,000 events were gated (Extended Data Fig. 5). Graphs show the percentage of γH2AX-positive cells as means ± s.d. from one of two independent experiments performed in triplicate. ^*^P = 0.0179, ^***^P < 0.001; one-way ANOVA (*F*_S(24h)_ = 4.937, *F*_S(48h)_ = 303.4, *F*_G2/M(24h)_ = 2.968, *F*_G2/M(48h)_ = 310, d.f. = 3). **d**, THP-1 cell protein levels of checkpoint and cell death markers on TH7299. β-Actin was used as a loading control. A representative experiment is shown (*n* = 2). **e**, Subcellular fractionation of THP-1 cells for replication markers on TH7299 (24 h, 10 μM). Chr., chromatin-bound fraction; Sol., soluble fraction (cytoplasmic and nuclear). Histone H3 and β-actin were used as loading controls for the chromatin-bound and soluble fractions, respectively. A representative experiment is shown (*n* = 2). **f**, DNA fiber assay on TH7299 (3 μM), TH9619/MTX (50 nM) and VE-821 (1 μM) in HL-60 and THP-1 cells compared with nontumorigenic LCL-534 and LCL-889 cells. Scale bar, 5 μm. Scatter dot plots show the mean and distribution of RF speeds for each treatment. From left to right: *n* (fibers per condition) = 198, 227, 244, 182, 255, 201, 278, 461, 240, 219, 229, 223, 152, 250, 165, 176, 344, 183, 447 and 326. A representative of two independent experiments is shown. ^***^P < 0.001; one-way ANOVA with Holm–Šídák correction for multiple comparisons (*F*_HL-60_ = 233.9, *F*_THP-1_ = 187, *F*_LCL-534_ = 165.3, *F*_LCL-889_ = 435.1, d.f. = 4). Source data

**Extended Data Fig. 5:**
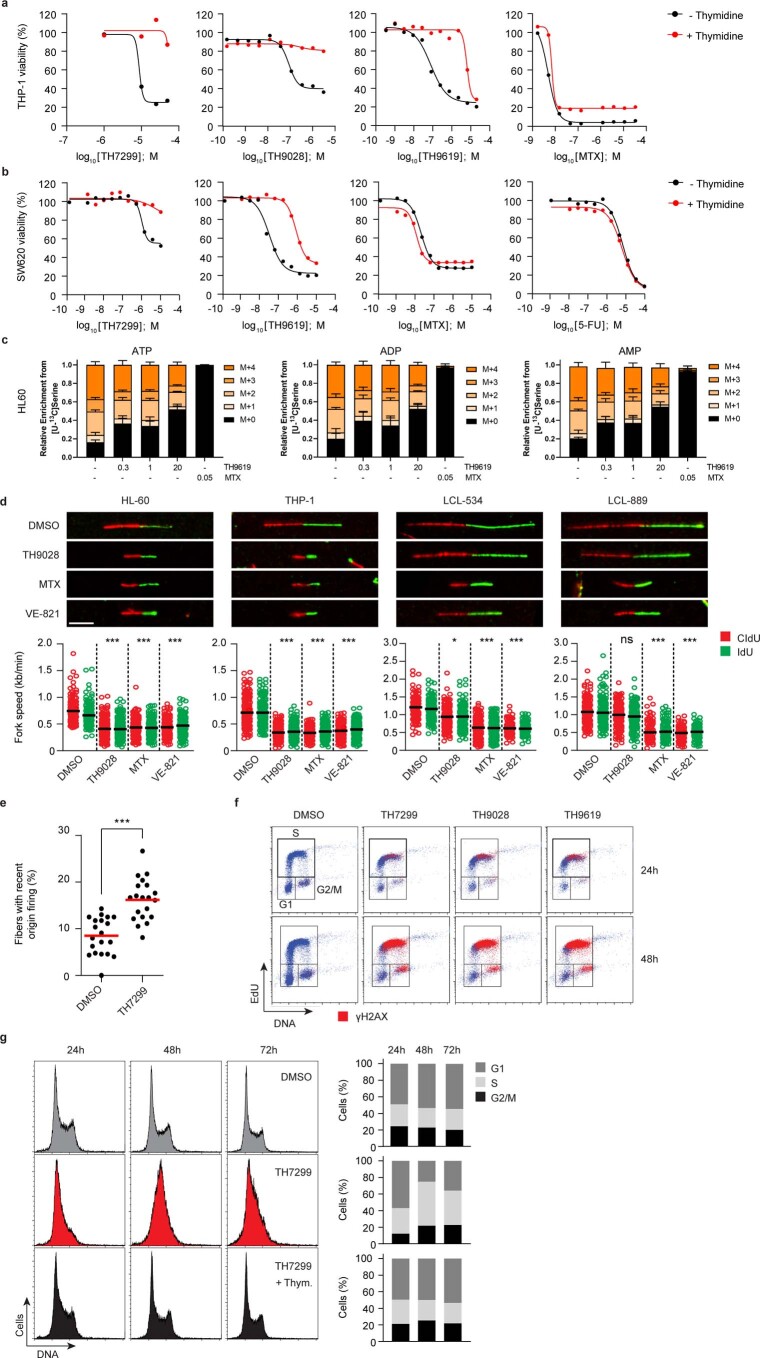
MTHFD2i induce replication stress selectively in cancer cells, rescuable by thymidine. (a) THP-1 cell viability upon MTHFD2i or methotrexate (MTX) ± thymidine 50 μM (96 h). Data from one representative experiment are displayed as means, n = 2. (b) SW620 cell viability upon TH7299, TH9619, MTX or 5-fluorouracil (5-FU) ± thymidine 50 μM (96 h). Data from one representative experiment are displayed as means, n = 2. (c) TH9619 reduces serine-derived carbon-flux into purine synthesis. Graphs show mass isotopologue distribution (MID) of intracellular ATP, ADP, and AMP upon [U-^13^C]-serine tracer in response to 24 h treatment with 0.3, 1, or 20 μM TH9619 or 50 nM MTX in HL-60 cells; mean ± SEM of three independent experiments each measured in triplicate. wells. Reduced labelling from [U-^13^C]-serine of ATP, ADP, and AMP as well as increased relative percentage of unlabeled M + 0 isotopologues of ATP, ADP, and AMP is indicative of a reduced flux of ^13^C-carbon units from serine to purine synthesis upon TH9619 treatment. MTX serves as a positive control for purine synthesis inhibition. (d) DNA fiber assay upon TH9028 50 nM, MTX 50 nM and VE-821 1 μM in AML and non-malignant cells. Scale bar, 5 μm. Dotplots show the distribution and calculated mean of replication fork speed per treatment. From left to right, n (fibers/condition) = 165; 345; 192; 227; 220; 284; 237; 279; 142; 192; 218; 171; 192; 170; 114; 248. Shown is a representative out of two independent experiments. * P < 0.05, *** P < 0.001; one-way ANOVA with Dunnett’s multiple comparison test (F_HL-60_ = 492.6, F_THP-1_ = 771.6, F_LCL-534_ = 624, F_LCL-889_ = 770.6, DF = 3). (e) Replication origin firings upon TH7299 (10 μM, 24 h) in THP-1 cells. Data are displayed as means ± SD, n = 20. *** P < 0.001; one-way Mann-Whitney rank test (F = 39, DF = 1). (f) Cell cycle analysis of DNA damage (γH2AX) induction upon MTHFD2i treatment in HL-60 cells. Per sample, at least 15,000 events were gated. (g) Thymidine rescue of cell cycle progression upon TH7299 treatment in THP-1 cells. At least 10,000 events were analyzed/condition. Bar graphs represent the percentage of cells in each phase, displayed as means, n = 2. Source data

As inhibition of viability induced by MTHFD2 siRNA could be rescued with the addition of thymidine (Fig. 1c), we next assessed whether viability of MTHFD2 inhibitor-treated cells could also be rescued by thymidine supplementation. Consistent with the knockdown data, thymidine supplementation completely rescued the viability defects in MTHFD2 inhibitor-treated HL-60 cells, a trend also observed in THP-1 AML cells and SW620 colorectal cancer cells (Fig. 4a and Extended Data Fig. 3). In contrast, the proliferation block induced by antifolates could not be rescued by increased thymidine levels. Furthermore, employing [U-^13^C]serine tracing^28^ to explore one-carbon flux, we revealed that TH9619 reduces purine synthesis from serine, but does not cause the complete block observed with MTX in HL-60 cells (Extended Data Fig. 5). Collectively, our data indicate that MTHFD2 inhibitors have a distinct mechanism of action to existing antifolates.

To validate the effects of MTHFD2 inhibitors on DNA replication, we performed DNA fiber assays in THP-1 cells together with thymidine supplementation (Fig. 4b). Treatment with TH7299 resulted in approximately 50% reduction of RF speed and significantly shorter DNA fibers; meanwhile supplementation with thymidine was sufficient to fully rescue replication speeds. Similar fork speed reductions were also observed on TH9028 treatment in HL-60 and THP-1 cells (Extended Data Fig. 5). DNA fiber analysis of TH7299-treated cells also revealed an increase in replication origin firing (Extended Data Fig. 5), an early response to RS as cells attempt to compensate for slow forks by firing additional replication origins^29–31^.

We performed cell-cycle analysis in combination with γH2AX staining in HL-60 cells on treatment with MTHFD2 inhibitors and observed a time-dependent accumulation of cells with DNA damage in the S phase, visible after 24 h but most prominent after 48 h, with almost 60% of cells in the S phase being positive for γH2AX at this timepoint (Fig. 4c and Extended Data Fig. 5). Moreover, cell-cycle analysis on MTHFD2 inhibitors over time also confirmed this progressive accumulation of cells in the S phase coinciding with the induction of DNA damage, which was reversed by thymidine supplementation (Extended Data Fig. 5).

To test whether MTHFD2 inhibitors trigger the DDR and apoptosis, we followed the protein expression levels and phosphorylation status of different DDR and cell death markers. After 24 h, we observed a marked increase in the phosphorylation of mammalian replication protein A (RPA) and Chk1, indicative of single-stranded DNA (ssDNA) regions and activation of the ATR-mediated DDR pathway (Fig. 4d). A gradual expression of p21 (inactivating CDK2) and accumulation of inactivating phosphorylation of CDK1 also occurred. The timing of these events corresponded to the cell cycle and flow cytometry DDRs, altogether suggesting that MTHFD2 inhibitor-induced RS triggers cell-cycle arrest in the S and G2/M phases of the cell cycle. At later timepoints, increased levels of γH2AX, cleaved caspase and cleaved PARPs confirmed induction of apoptosis in these cells. Finally, using subcellular fractionation after 24 h of MTHFD2 inhibitors, we found higher levels of free Mcm6, proliferating cell nuclear antigen (PCNA) and DNA Polδ, as well as an increase in chromatin-bound RPA subunits (Fig. 4e), further suggesting the formation of ssDNA after the collapse of RFs and the release of replisome components on MTHFD2 inhibitors^31^.

To assess the cancer specificity of the replication defects caused by MTHFD2 inhibitors, we treated AML and nontumorigenic LCL cells with TH7299, TH9619, MTX or the ATR inhibitor VE-821. Although MTX and VE-821 significantly blocked DNA RF progression in both cancer and nontumorigenic cells, MTHFD2 inhibitors effectively impaired DNA replication only in AML cells, sparing nontumorigenic lymphocytes (Fig. 4f). The same cancer selectivity was observed for TH9028 (Extended Data Fig. 5). Thus, our data suggest that MTHFD2 activity is essential for proper and correct completion of DNA replication specifically in tumor cells.

### MTHFD2 inhibitors cause uracil misincorporation into DNA

**Fig. 5:**
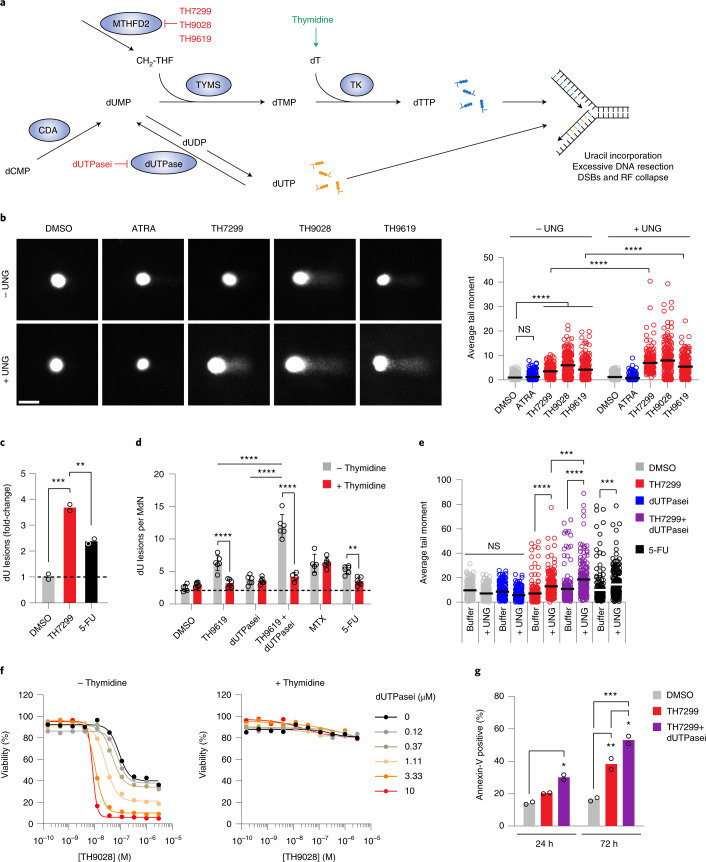
MTHFD2 inhibitors exacerbate uracil misincorporation into DNA. **a**, Proposed mechanism for the antitumor effect of MTHFD2 inhibitors via thymine-less-induced RS. MTHFD2 supports de novo thymidylate synthesis by providing CH_2_-THF. On MTHFD2 inhibition, accumulation of dUMP promotes uracil misincorporation, causing DNA damage. Failing to repair these lesions, cells undergo RF collapse and cell death. A combination of MTHFD2 and dUTPase inhibitors further increases uracil misincorporation and apoptosis. Thymidine supplementation bypasses this, rescuing DNA replication and cell viability. CDA, cytidine deaminase; DSBs, double-strand breaks; dUTPasei, dUTPase inhibitor; TK, thymidine kinase. **b**, Comet assay in THP-1 cells. Scale bar, 2 μm. Dot plots represent comet tail moment per cell and bars display the mean (*n* = 200 cells per condition). ^****^P < 0.0001; one-way Kruskal–Wallis test with Dunn’s multiple comparison correction (*F* = 904.2, d.f. = 9). **c**, Genomic uracil incorporation on TH7299 in THP-1 cells (10 μM, 48 h). Bars represent dU lesions as fold-change over DMSO displayed as means (*n* = 2). One of two independent experiments is shown. ^**^P < 0.01, ^***^P < 0.001; one-way ANOVA (*F* = 156.4, d.f. = 2). **d**, Genomic uracil incorporation in HL-60 cells (TH9619 100 nM ± dUTPase inhibitor 10 µM, MTX 100 nM, 5-FU 10 μM ± thymidine 10 μM, 48 h). Bars represent dU lesions per million deoxynucleotides (MdN). Data are displayed as pooled means ± s.d. from two independent experiments performed in triplicate. ^**^P = 0.0063, ^****^P < 0.0001; two-way ANOVA with Holm–Šídák correction for multiple comparisons (*F*_treatment_ = 48.64, *F*_thymidine_ = 87.47, d.f._treatment_ = 5, d.f._thymidine_ = 1). **e**, Comet assay in THP-1 cells on TH7299 ± dUTPase inhibitor (10 μM, 24 h). Dot plots represent tail moment per cell and bars display the mean (*n* = 200 cells per condition). ^***^P < 0.001, ^****^P < 0.0001; one-way Kruskal–Wallis test with Dunn’s multiple comparison correction (*F* = 361.4, d.f. = 9). **f**, THP-1 cell viability on TH9028–dUTPase inhibitor combination ± thymidine 10 μM at 72 h. One of three independent experiments is shown. Data are displayed as means (*n* = 2). **g**, Apoptosis analysis in THP-1 cells. Approximately 15,000 events per condition were analyzed. Bars represent annexin-V-positive populations as means (*n* = 2). ^*^P_24h_ = 0.0151, ^*^P_72h_ = 0.0213, ^**^P = 0.0029, ^***^P = 0.0002; one-way ANOVA (*F* = 43.89, d.f. = 5). One of two independent experiments is shown. Source data

Unlike other deoxynucleotide triphosphates (dNTPs), which are formed from their diphosphate precursors, deoxythymidine triphosphate (dTTP) is formed from deoxythymidine monophosphate (dTMP), which can be synthesized de novo only from deoxyuridine monophosphate (dUMP)^32^ (Fig. 5a). Both thymidine and uracil can form stable base-pairs with adenine, and most DNA polymerases cannot distinguish between the two, readily incorporating either molecule depending solely on their relative abundance^33^. To prevent uracil misincorporation into DNA and genomic instability, the dUTP:dTTP ratio is tightly regulated and kept very low (<0.03) to promote correct DNA synthesis^34,35^. Thymidine can be used by the cells to generate dTTP through the concerted actions of thymidine kinase, thymidylate kinase and nucleoside diphosphate kinase^36^. As we have shown in the present study, thymidine supplementation completely rescued the metabolic loss of MTHFD2 activity in DNA replication and proliferation assays. This prompted us to question whether MTHFD2 inhibitors led to an imbalanced dUTP:dTTP pool causing excessive uracil misincorporation into DNA (Fig. 5a). To address this, we performed a modified comet assay with enzymatic cleavage of genomic uracil using uracil DNA glycosylase (UNG) on MTHFD2 inhibitor treatment. Already, after 16 h, MTHFD2 inhibitor-treated cells displayed increased tail moments, corresponding to increased uracil incorporation (Fig. 5b). Also, an almost fourfold increase of genomic uracil was detected by mass spectrometry (MS) analysis in the MTHFD2 inhibitor-treated cells compared with control treatment, which was significantly higher compared with the positive control treatment with 5-FU (Fig. 5c,d). Thymidine supplementation restored normal genomic uracil levels on MTHFD2 inhibitor and 5-FU treatment, which was not the case for MTX (Fig. 5d). This is consistent with our data showing that decreased viability on MTX and PMTX treatments cannot be rescued by thymidine supplementation (Fig. 4a). Thus, MTHFD2 inhibitors promote excessive uracil DNA misincorporation during replication, most probably by creating an imbalance in the dUTP:dTTP pool, resulting in RS.

One of the main mechanisms to control the dUTP:dTTP balance relies on the activity of dUTPase, an enzyme that hydrolyzes dUTP to dUMP and pyrophosphate^37^. Thus, dUTPase activity prevents uracil misincorporation into DNA, and provides dUMP for de novo thymidylate synthesis. In the present study, we measured genomic uracil incorporation into HL-60 and THP-1 cells on MTHFD2 inhibitors alone or in combination with a dUTPase inhibitor (compound 1 (ref. ^38^), compound 12k^39^). In line with our proposed mechanism of action, combination of MTHFD2 inhibitors and dUTPase inhibitors increased DNA uracil lesions and comet tail moment (Fig. 5d,e). We also hypothesized that, by inhibiting dUTPase in MTHFD2 inhibitor-treated cells, we would deplete them of both the dUMP and CH_2_-THF substrates for thymidylate synthase (Fig. 5a). Accordingly, dUTPase inhibitors synergized with MTHFD2 inhibitors in a dose-dependent manner to reduce cell viability and increase apoptosis in THP-1 cells (Fig. 5f,g). In these experiments, viability could also be entirely restored by thymidine supplementation (Fig. 5f).

**Extended Data Fig. 6:**
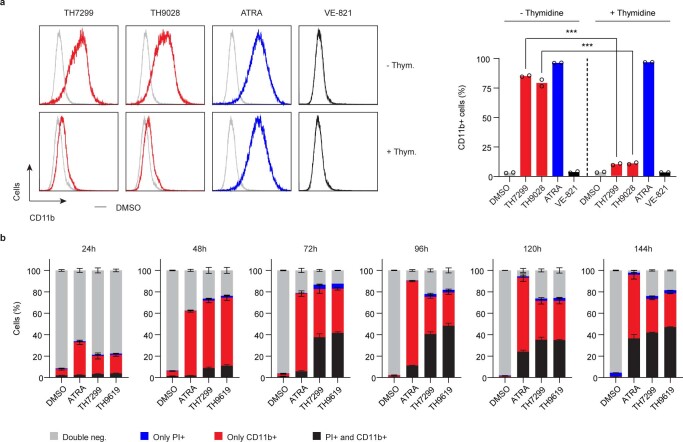
MTHFD2i induce differentiation of AML blasts. (a) Flow cytometry analysis of CD11b expression in HL-60 cells after 96 h incubation with TH7299 3 μM, TH9028 50 nM, ATRA 1 μM, or ATRi VE-821 1 μM, in presence or absence of thymidine 5 μM supplementation. To the left, representative histograms, and to the right, bar graphs showing the percentage of CD11b positive cells, displayed as means, n = 2. *** P < 0.001; one-way ANOVA (F = 1983, DF = 9). Shown is a representative out of two independent experiments. (b) CD11b/PI live staining of HL-60 cells upon incubation with ATRA 1 μM, TH7299 5 μM or TH9619 50 nM, assessed at each indicated time point. Approximately 20,000 events were gated per condition. Bar graphs represent the percentage of double-negative, single positive and double-positive populations as means ± SD. Shown is a representative experiment out of two independent experiments conducted in triplicate. Source data

Moreover, we noticed that approximately 20% of the responding cancer cells consistently survived despite increasing concentrations of MTHFD2 inhibitors (Fig. 3a), which could be related to earlier observations that MTHFD2 knockdown induces AML differentiation^19^. In line with this, we found that MTHFD2 inhibitors increased expression of the myeloid differentiation marker CD11b surface integrin to an extent comparable with the differentiation agent ATRA, which could also be rescued by thymidine supplementation (Extended Data Fig. 6).

### MTHFD2 inhibitors sensitize cancer cells to ATR-signaling blockade

**Extended Data Fig. 7:**
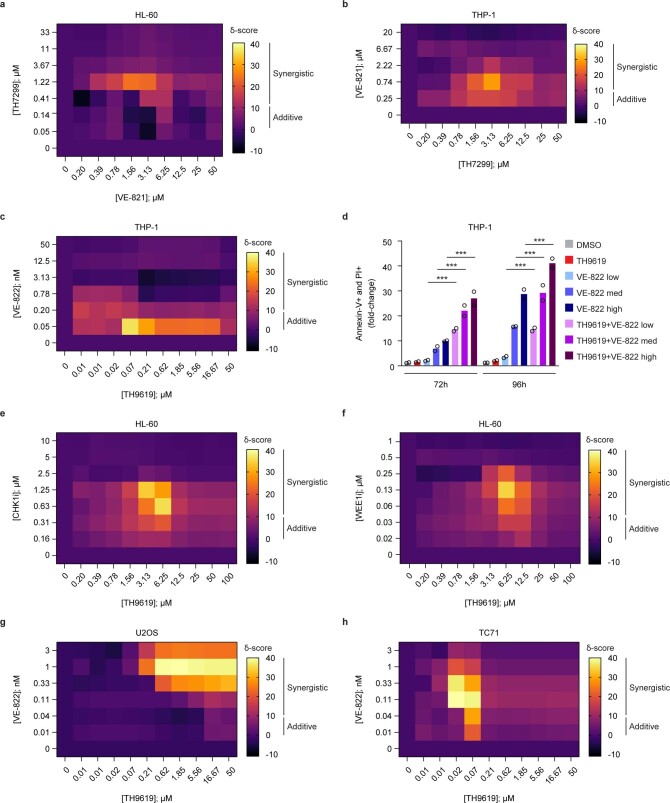
MTHFD2i sensitize cancer cells to ATR signaling blockade. (a, b) Synergy score matrices for HL-60 (a) and THP-1 (b) cells following 72 h of combined TH7299 and ATRi VE-821 treatment. Normalized cell viability results were used to calculate synergy matrix heat-maps to visualize the interaction landscape for each drug pair using the SynergyFinder zero-interaction potency (ZIP) score model with baseline correction. Synergy scores (δ-scores) between 0 and 10 suggest the interaction between the two drugs is likely additive, while synergy scores larger than 10 indicate the interaction between the two drugs is probably synergistic. Shown is a representative out of two independent experiments. (c) Synergy score matrix for THP-1 cells following 96 h of combined TH9619 and ATRi VE-822 treatment. Shown is a representative out of two independent experiments. (d) Annexin-V/PI flow cytometry analysis of apoptosis in THP-1 cells upon TH9619 50 nM treatment, alone or in combination with low (100 nM), medium (250 nM) or high (500 nM) doses of ATRi VE-822, for 72 h or 96 h. Approximately 20,000 events were analyzed per condition. Shown is the quantification of annexin-V and PI double-positive apoptotic populations as fold-change over DMSO control, bars represent means, n = 2. *** P < 0.001 (72 h low VE-822-TH9619 + VE-822 P = 0.0002; 96 h low VE-822-TH9619 + VE-822 P = 0.0007; 96 h high VE-822-TH9619 + VE-822 P = 0.0002); two-way ANOVA analysis (F_time_ = 85.52, F_treatment_ = 153.2, DF_time_ = 1, DF_treatment_ = 7). This is a representative out of four independent experiments. (e, f) Synergy score matrices for HL-60 cells following 96 h of combined TH9619 and CHK1i (CCT245737) (e), or combined TH9619 and WEE1i (MK-1775) (f) treatment. Shown is a representative out of two independent experiments. (g, h) Synergy score matrix for U2OS and TC71 cells following 96 h of combined TH9619 and ATRi VE-822 treatment. Shown is a representative out of two independent experiments. Source data

Inhibitors of ATR or Chk1 are currently being evaluated as anticancer treatments in cancers harboring high levels of RS, such as hematological cancers^40–43^. Given that MTHFD2 inhibitors induce RS with increased selectivity in transformed cells, we hypothesized that ATR inhibitors would potentiate their antitumor efficacy. In accordance with this, we found that MTHFD2 inhibitors TH7299 and TH9619 are synergistic when combined with ATR inhibitors VE-821 or VE-822 in both AML HL-60 and THP-1 cells, as well as in bone cancer cell lines U2OS and TC71, increasing apoptosis in a time- and dose-dependent manner (Extended Data Fig. 7). In addition, we tested Chk1 inhibitor CCT245737 and Wee1 inhibitor MK-1775, which also synergized with TH9619 in HL-60 cells (Extended Data Fig. 7). Collectively, these results confirm the proposed mechanism of action for MTHFD2 inhibitors via induction of RS and provide rational support for the potential future use of MTHFD2 inhibitors as cancer-selective sensitizers to ATR-signaling blockade therapies.

### MTHFD2 inhibitor TH9619 impairs cancer progression in vivo

**Extended Data Fig. 8:**
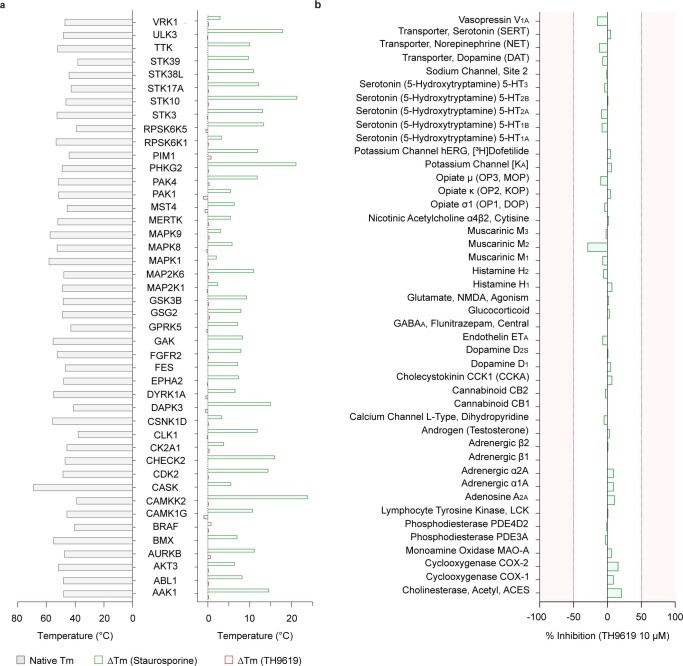
In vitro selectivity and safety evaluation of TH9619. (a) DSF-based selectivity screen of TH9619 12 µM against a curated library of 44 kinases, using staurosporine as a positive control. Bars represent the mean change in protein melting temperature (ΔTm) of one experiment performed in triplicate. (b) Selectivity of TH9619 10 µM against the SafetyScreen44 panel from Eurofins. Bars represent mean inhibition in percentage of one experiment performed in duplicate. Source data

TH9619 shows very good solubility and metabolic stability in combination with low plasma protein binding (Supplementary Table 4), but also poor passive cell permeability that is probably explained by the low lipophilicity (cLog*P*: −2.25) and large polar surface area (239 Å^2^ (23.9 nm)), suggesting active transport over membranes, probably via folate transporters and/or receptors. No inhibition by TH9619 was observed on 44 kinases and the Eurofins SafetyScreen44 panel, suggesting high target selectivity (Extended Data Fig. 8). Furthermore, TH9619 was comparably potent against mouse MTHFD2 (half-maximal inhibitory concentration (IC_50_) of 66 nM), indicating that mice could be used as a model for safety and tolerability studies.

**Fig. 6:**
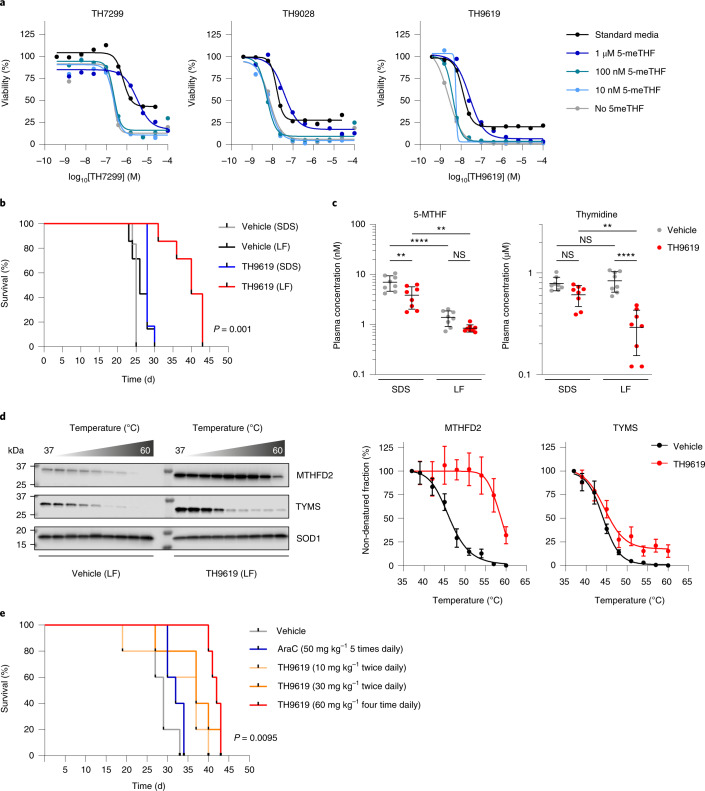
MTHFD2 inhibitor TH9619 impairs cancer progression in vivo. **a**, Cell viability of HL-60 cells on TH7299, TH9028 and TH9619 treatment for 96 h under increasing concentrations of medium folate supplementation. Representatives of three independent experiments are shown. Data are displayed as means (*n* = 2 independent cell cultures). **b**, Kaplan–Meier curve showing overall survival of NOG mice with HL-60 IV xenograft tumors after treatment with TH9619 versus vehicle control, on standard chow (SDS) or LF (*n* (SDS groups) = 6 mice, *n* (LF groups) = 7 mice). *P* = 0.001 calculated using a one-way Mantel–Cox log-rank test. **c**, Plasma concentration of 5-MTHF and thymidine at the time of sacrifice. Data are displayed as median ± s.d. (*n* = 8 mice per group). ^**^P_5-MTHF(SDS)_ = 0.0015, ^**^P_5-MTHF(TH9619 SDS-LF)_ = 0.0026, ^**^P_thymidine_ = 0.0013, ^****^P < 0.0001; two-way ANOVA with Tukey’s correction for multiple comparisons (*F*_5-MTHF(diet)_ = 63.93, *F*_5-MTHF(treatment)_ = 11.82, *F*_thymidine(diet)_ = 6.307, *F*_thymidine(treatment)_ = 46.01, d.f._diet_ = 1, d.f._treatment_ = 1); NS, not significant. **d**, Target engagement of MTHFD2 and TYMS in tumor samples analyzed using CETSA. Representative images are shown for one of two independent experiments. Graphs show the nondenatured target fraction from TH9619-treated animals and vehicle controls from the LF group, displayed as means ± s.d. (*n* = 4). MTHFD2 melting temperature (Δ*T*_m_) = 17 °C, TYMS Δ*T*_m_ = 1 °C. **e**, Kaplan–Meier curve showing overall survival of NSG mice on LF with HL-60 IV xenograft tumors after treatment with TH9619 10 mg kg^−1^ twice daily, TH9619 30 mg kg^−1^ twice daily, TH9619 60 mg kg^−1^ four times daily, AraC 50 mg kg^−1^ five times daily or vehicle control (n = 5 mice per group). There was significantly improved survival in the TH9619 group receiving 60 mg kg^−1^ four times daily compared with the AraC and vehicle groups. *P* = 0.0095 calculated using a one-way Mantel–Cox log-rank test. Source data

**Extended Data Fig. 9:**
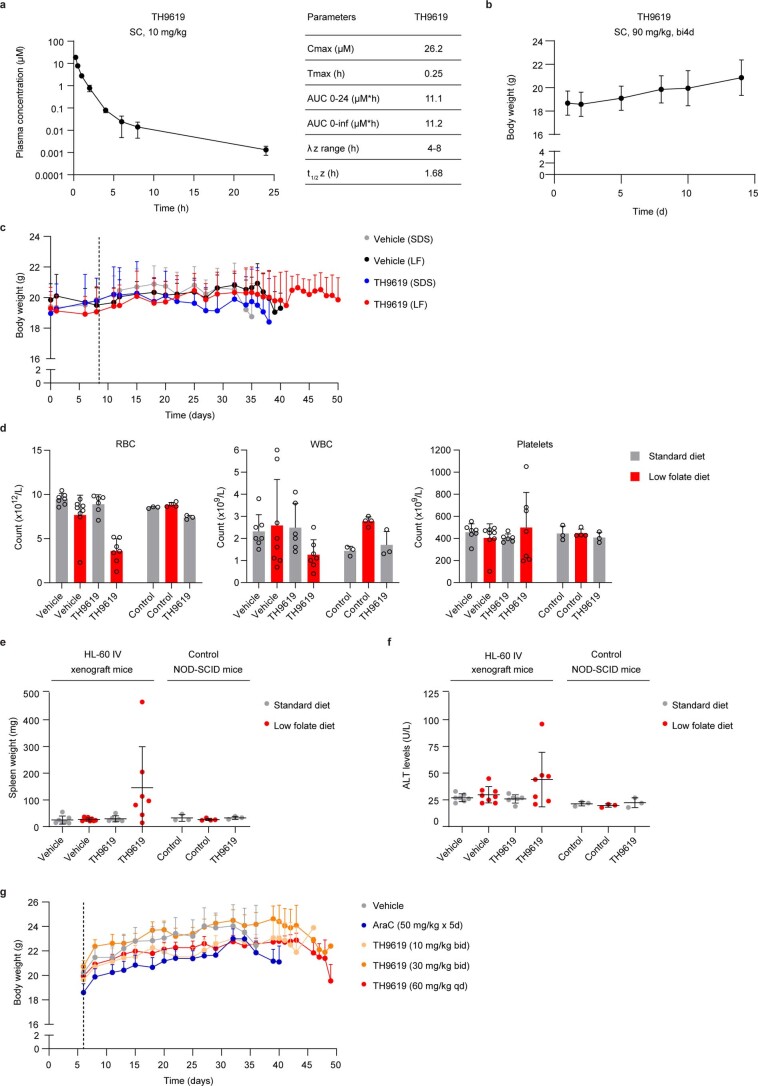
In vivo profiling of TH9619 in mouse models. (a) Plasma concentration and pharmacokinetic (PK) evaluation of TH9619 following subcutaneous administration of 10 mg/kg in female NOG mice. Data are displayed as means ± SD, n = 3. (b) Dose tolerability study after repeated subcutaneous administrations of TH9619 in female NOD-SCID mice. TH9619 90 mg/kg was administered twice daily on four occasions (twice per week for two weeks). Body weight data are displayed as means ± SD, n = 4. (c) Body weight measurements for animals included in the HL-60 xenograft efficacy study. Data are displayed as means + SD, n (mice/group) = 7 (vehicle SDS), 8 (vehicle LF), 6 (TH9619 SDS), 7 (TH9619 LF). Dotted line indicates treatment initiation on day 11. (d) Summary of hematology parameters upon sacrifice of HL-60 xenograft efficacy study animals and satellite control mice. Total count of red blood cells (RBC), white blood cells (WBC) and platelets per liter are displayed as means + SD. From left to right, n (mice/group) = 7; 8; 6; 7; 3; 4; 3. (e) Spleen weight upon sacrifice of HL-60 xenograft efficacy study animals and satellite control mice. Data are displayed as means ± SD. From left to right, n (mice/group) = 7; 8; 6; 7; 3; 4; 3. (f) Plasma concentration of alanine transaminase (ALT) upon sacrifice of HL-60 xenograft efficacy study animals and satellite control mice. Data are displayed as means ± SD. From left to right, n (mice/group) = 7; 8; 6; 7; 3; 4; 3. (g) Body weight measurements for animals included in the HL-60 xenograft efficacy study comparing to standard of care cytarabine (AraC). Data are displayed as means + SD, n = 5 per group. Dotted line indicates treatment initiation on day 6. Source data

**Extended Data Fig. 10:**
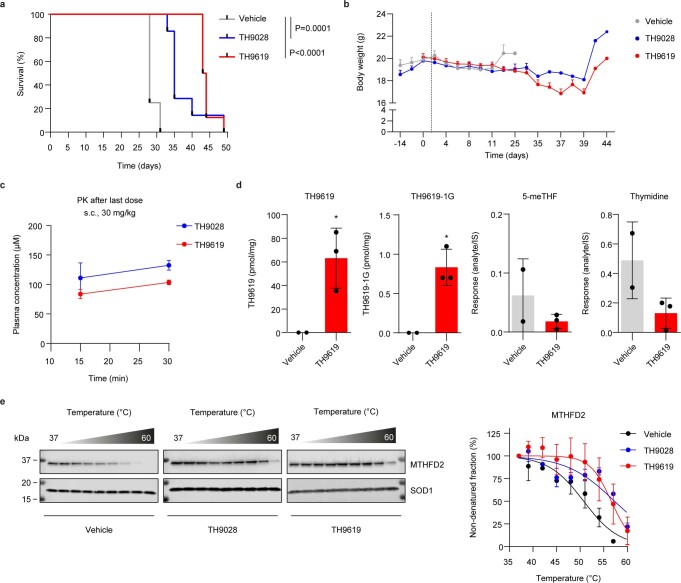
In vivo profiling of TH9028 and TH9619 in mouse models. (a) Kaplan-Meier curve showing overall survival of NOG mice with HL-60 IV xenograft tumors after treatment with TH9028 (30 mg/kg bid) or TH9619 (30 mg/kg bid) versus vehicle control, on low folate diet, n = 6–7 per group. P = 0.001 calculated using a one-way Mantel-Cox log-rank test. (b) Body weight measurements. Mouse body weight was monitored for two weeks before treatment start and followed throughout the study. At day 37 of treatment the remaining animals regained weight after diet shift from low folate diet to standard chow diet. Data are displayed as means + SEM, n = 7 (TH9028) or 8 (vehicle/TH9619) per group. Dotted line indicates treatment initiation on day 1. (c) Plasma concentration levels at 15 min and 30 min after the last administration of TH9028 (30 mg/kg bid) or TH9619 (30 mg/kg bid) in xenograft mice, n = 3 per group. Data are displayed as means ± SD. (d) Intratumor levels of TH9619, monoglutaminated TH9619 (TH9619-1G), 5-meTHF and thymidine at the time of sacrifice, measured by LC-MS and displayed as means ± SD, n = 2 (vehicle) or 3 (treated). * P < 0.05 calculated by unpaired two-tailed t-test (P_TH9619_ = 0.0449, t_TH9619_ = 3.324, DF_TH9619_ = 1; P_TH9619-1G_ = 0.0168, t_TH9619-1G_ = 4.841, DF_TH9619-1G_ = 1). (e) Target engagement of MTHFD2 in tumor samples analyzed using CETSA. Graphs show the non-denatured target fraction from TH9028- and TH9619-treated animals and vehicle controls, displayed as means ± SD, n = 2 (vehicle) or 3 (treated). MTHFD2 ΔTm (TH9028) = 6.4 °C, MTHFD2 ΔTm (TH9619) = 5.9 °C. Source data

We next sought to determine the tolerability, pharmacokinetic properties and efficacy of TH9619 in a mouse disease model of AML. Subcutaneous (s.c.) administration of 10 mg kg^−1^ of TH9619 resulted in rapid systemic absorption, a maximum plasma concentration (*C*_max_) of 26 µM and a half-life of 1.7 h (Extended Data Fig. 9). Moreover, TH9619 was well tolerated with no significant decrease in body weight or any observable behavioral changes on repeated s.c. administration of 90 mg kg^−1^ in mice on standard chow (SDS) for up to 2 weeks (Extended Data Fig. 9). The pronounced difference in folate and thymidine levels between mice and humans^44^ represented a major concern, because the approximately 100-fold higher thymidine levels in mice could rescue the antiproliferative effects of MTHFD2 inhibitors. In cell culture, decreasing the folate levels in the medium resulted in increased MTHFD2 inhibitor potency (Fig. 6a). To reduce the folate levels in mice, they were fed a low-folate diet (LF) for 2 weeks before the start and throughout the duration of the survival study. A xenograft AML model was established in 7-week-old NOG mice by intravenous injection of HL-60 cells, allowing the formation of tumors over 11 d. The four tested groups consisted of mice on standard chow or LF, receiving either vehicle or s.c. TH9619 (30 mg kg^−1^, twice daily). TH9619 combined with the LF significantly prolonged mouse survival by a median of 2 weeks compared with vehicle without affecting body weight (Fig. 6b, and Extended Data Figs. 9 and 10). Consistent with the proposed mechanism of action, systemic thymidine levels were significantly lower in both diet treatments, but particularly in the LF TH9619-treated mice (Fig. 6c and Extended Data Fig. 10).

Furthermore, we observed MTHFD2 target engagement with TH9619 in vivo by performing CETSA on tumor samples (Fig. 6d and Extended Data Fig. 10), whereas no stabilization could be observed in the vehicle-treated animals, or those on TYMS with TH9619.

Next, we compared the efficacy of TH9619 with the standard of care for AML, AraC. A dose-dependent improvement of survival occurred on administration of TH9619, with TH9619 60 mg kg^−1^ four times daily significantly outperforming AraC (Fig. 6e). Notably, animals in the AraC treatment group had consistently lower body weight compared with the other groups, showing that therapeutic doses of TH9619 featured reduced in vivo toxicity compared with AML standard-of-care chemotherapy (Extended Data Fig. 9). Thus, we conclude that TH9619, a first-in-class small-molecule MTHFD2 inhibitor, displays notable anticancer activity in vivo with a favorable profile for clinical use.

## Discussion

In the present study, we present new MTHFD2 inhibitors that selectively kill cancer cells through thymine-less death, representing a promising therapeutic approach in cancer. We confirm previously published cancer dependency on MTHFD2 for cancer cell proliferation using an RNAi approach^15,19^, and show that WT, but not catalytically dead, RNAi-resistant MTHFD2 can rescue cytotoxicity on MTHFD2 silencing. Although this implies that the catalytic activity of MTHFD2 is required for cancer cell growth, conflicting reports of viable CRISPR–Cas9-mediated MTHFD2 knockout cells^45^ and larger CRISPR–Cas9 screening datasets suggest that MTHFD2 is mostly nonessential in cancer cells^46–49^. The discrepancy between CRISPR–Cas9 screens and RNAi data reported by us and others is probably a result of the high plasticity of metabolic pathways^50^. Hence, although the MTHFD2-dependent mitochondrial pathway to generate formate is the default route in most cancer cells, the cells can probably switch to use cytosolic pathways under certain conditions, such as survival selection pressure on MTHFD2 deletion by CRISPR–Cas9. In support of this notion, it has been demonstrated that the normally nonessential SHMT1 becomes essential in an MTHFD2 knockout background^45^.

In the present study, we describe first-in-class, low-nanomolar inhibitors targeting the THF-binding pocket of MTHFD2. These MTHFD2 inhibitors are largely selective, engage with the target in cells and tumors, and effectively block proliferation of AML cancer in vitro and in vivo, with a therapeutic window spanning several orders of magnitude. Given the known plasticity of this metabolic pathway, it may be advantageous that our lead compounds, including TH9619, target MTHFD2 and MTHFD2L, as well as MTHFD1 in parallel, prevent emerging resistance. It is interesting that, although our MTHFD inhibitors also enzymatically inhibit MTHFD2L and the DC domain of MTHFD1, the cancer-selective properties observed following MTHFD2 siRNA depletion remain the same using the MTHFD2 inhibitors, which could potentially be due to the relatively low expression levels of MTHFD1 compared with MTHFD2 in cancer cells. We also show that CRISPR–Cas9 *MTHFD2*^−*/*−^ knockout cancer cells and clones are significantly more resistant to TH9619 compared with the parental WT cells, demonstrating that inhibition of MTHFD2 is necessary for the efficacy of TH9619 in cancer cells.

Moreover, we determine in detail the mechanism of action of MTHFD2 inhibition. We show that both MTHFD2 RNAi knockdown and MTHFD2 inhibitors kill cancer cells through depletion of thymidine, resulting in misincorporation of uracil and RS, which induces cell death in cancer, but not in nontumorigenic cells. We propose that MTHFD2 is crucial for sustaining the production of CH_2_-THF, generated as a substrate for TYMS in cancer cells. Also, we propose that the MTHFD2 inhibitor compounds described in the present study probably have a single mechanism of action because the cytotoxicity is rescued over three orders of magnitude simply by adding thymidine. The primary cause for toxicity is related to thymidine loss, although this does not exclude some contribution from other functions of MTHFD2 outside of thymidine production, including purine synthesis. There could also be potential off-target effects of the MTHFD2 inhibitors at higher concentrations, which should be tested in future studies.

We investigated the proposed mechanism of action further by removal of dUMP, required for TYMS for thymidylate synthesis, by dUTPase inhibition, and found a strong synergy with MTHFD2 inhibitors. The complete rescue of the MTHFD2 inhibitor–dUTPase inhibitor combination with thymidine, as well as the increase in dUTP incorporation during DNA replication, provide further support for the model and the distinct mechanism of action proposed in the present study.

Intriguingly, the metabolic salvage of MTHFD2 inhibitor toxicity with thymidine rescue was not observed in the case of classic antifolates that largely target the same pathways. The reason for the poor metabolic rescue of antifolates is probably due to their polypharmacology and diverse mechanisms of action. The specific mechanism of action of MTHFD2 inhibitors presumably also explains their high tolerability and lack of general efficacy across many cancer cell lines. Although high tolerability is positive, the lack of general efficacy makes translation more difficult in contrast to antifolates, for instance, and a biomarker-led approach is probably needed to predict sensitive cancers in the clinic. In addition to this, we show that preclinical in vitro and in vivo research of MTHFD2 inhibitors is highly influenced by folate and nucleoside levels in the medium or plasma of mice, making in vitro predictions of clinical effects a challenge. As the experimental conditions are important, it is likely that conflicting results on MTHFD2 inhibitors will appear in the literature that may even dispute the usefulness of MTHFD2 inhibitors altogether.

Although MTHFD2 inhibitors are mechanistically distinct from classic antifolates, they resemble DDR inhibitors (for example, ATR inhibitors, Chk1 inhibitors, PARP inhibitors) in causing RS. DDR inhibitors induce RS by targeting three distinct pathways: (1) DNA-damage signaling, (2) DNA repair or (3) DNA metabolism generating dNTPs^51^. As the RS phenotype is the predominant mechanism of action of MTHFD2 inhibitors, we would class them as DDR inhibitors, targeting DNA metabolism and thymidine synthesis. The synthetic lethal approach, for example, using PARP inhibitors in *BRCA1-* or *BRCA2-*mutated cancers^7,8^, has been the most successful path in the clinic for DDR inhibitors. In the present study, the differential cancer cell cytotoxicity suggests that a synthetic lethality approach may also be possible for MTHFD2 inhibitors, although the detailed genetic context has yet to be established. However, as MTHFD2 inhibitors cause cancer-specific replication stress, triggering the ATR-Chk1 signaling cascade, we observed a strong synergy between MTHFD2 inhibitors and ATR and Chk1 inhibitors. This synergy is unsurprising, because it is well established that both ATR and Chk1 inhibitors are required for survival under RS conditions^52^. As MTHFD2 inhibitors do not introduce RS in nontumorigenic cells, we propose that their combination with ATR or Chk1 inhibitors may improve the cancer selectivity and therapeutic index of these compounds. The strong synergy between MTHFD2 inhibitors and dUTPase inhibitors not only supports the mechanism of action, but may also be used as a strategy in the clinic. The dUTPase TAS-114 has been used in combination with a 5-FU derivative, S-1, in a phase II clinical trial, observing favorable tumor response and shrinkage trends, but failing to show an increase in progression-free survival and observing a 35% increase in grade 3 or greater toxicity, probably as a result of S-1 also being toxic to nontransformed cells^53^. We argue that an improved efficacy may be obtained without increase in adverse events when combining dUTPase inhibitors with well-tolerated MTHFD2 inhibitors.

It is well established that cellular proliferation rates and DNA replication demands inversely correlate with developmental stage^54^. MTHFD2 is expressed in embryonic tissue and during tissue maturation replaces MTHFD2L, among other metabolic changes, coinciding with a decrease in proliferation rates^17,18^. The differentiation of certain cell types can be especially dependent on the regulation of DNA replication^55^. In accordance with the observations using MTHFD2 RNAi^19^, our MTHFD2 inhibitors also induced AML differentiation and cancer cell death in a dose- and time-dependent manner. Together with its role in supporting elevated DNA synthesis levels in cancer cells^10,13,56^, MTHFD2 seems to provide a link between genome replication and maturation hindrance. In this regard, it is of interest that the ATR inhibitor VE-821 did not induce AML differentiation. This suggests that RS alone might not be sufficient to drive AML maturation on MTHFD2 inhibition, but rather that it depends on additional MTHFD2-specific mechanisms connecting the two processes. Clearly, these preliminary observations that MTHFD2 inhibitors induce differentiation require attention in future studies, especially in the context of tumors characterized by elevated numbers of cancer stem cells, where relapse is common.

In conclusion, we describe first-in-class potent MTHFD2 inhibitors targeting thymidine synthesis preferentially in tumor cells through a new mechanism of action targeting the DDR. We demonstrate MTHFD2 inhibitors being well tolerated and more efficient than standard of care in AML in-vivo models, and provide evidence of strong synergy with ATR inhibitors and dUTPase inhibitors that may improve current anticancer treatments.

## Methods

Our research complies with all relevant ethical regulations. All in vivo experiments in the present study were performed in accordance with the guidelines from the Swedish National Board for Laboratory Animals and the European Community Council Directive (86/609/EEC). Study protocols were approved by the Swedish Ethical Committee.

### Cell culture

LCL-534 and LCL-889 cells were obtained by immortalizing healthy donor B cells^57^. Normal, human, primary bone marrow CD34^+^ cells (American Type Culture Collection (ATCC), catalog no. PCS-800-012) were cultured in SFEM II medium (Stemcell) supplemented with a cytokine cocktail containing 20 ng ml^−1^ of each of the following cytokines: interleukin (IL)-6, IL-3, stem cell factor (R&D Systems), thrombopoietin and FLT-3/FLT-2 ligand (Stemcell). U2OS cells (ATCC, catalog no. HTB-96) were cultured in Dulbecco’s modified Eagle’s medium (DMEM) GlutaMAX medium (Life Technologies). HL-60 (ATCC, catalog no. CCL-240), THP-1 (ATCC, catalog no. TIB-202), MV4-11 (ATCC, catalog no. CRL-9591), CCRF-CEM (ATCC, catalog no. CCL-119), Jurkat (ATCC, catalog no. TIB-152), PL-21 (DSMZ, catalog no. ACC 536), LCL-534, LCL-889 and SW620 (ATCC, catalog no. CCL-227), and TC71 cells were cultured in RPMI 1640 GlutaMAX medium (Life Technologies). A SW620 *MTHFD2*^−*/*−^ cell pool was purchased from Synthego using a single-guide RNA sequence targeting exon 2 (5′-CGCCAACCAGGAUCACACUC-3′), and knockout confirmed by western blotting. CCD 841 cells were cultured in DMEM low-glucose medium supplemented with l-glutamine (Life Technologies). MCF10A cells (ATCC, catalog no. CRL-10317) were cultured in mammary epithelium basal medium supplemented with MEGM SingleQuots Supplements and Growth Factors (Lonza), 100 µg l^−1^ of cholera toxin (Sigma-Aldrich) and 7.5% heat-inactivated fetal bovine serum (FBS). U2OS cells stably transduced with Premo FUCCI Cell Cycle Sensor (Invitrogen) were established as previously described^58^ and cultured in McCoy’s 5A modified medium (Life Technologies). Unless specified otherwise, all media were supplemented with 10% heat-inactivated FBS, 100 U ml^−1^ of penicillin and 100 μg m^−1^ of streptomycin (Life Technologies). Cells were maintained at 37 °C with 5% CO_2_ in a humidified incubator. Cell lines were routinely tested for *Mycoplasma* contamination using the MycoAlert Mycoplasma Detection Kit (Lonza). None of the cell lines used in this study is listed in the International Cell Line Authentication Committee register of commonly misidentified cell lines.

### Plasmid construction for MTHFD2 mutants

Human MTHFD2 complementary (c)DNA with point mutations to confer resistance against three MTHFD2 siRNA oligonucleotides (J-009349-10, J-009349-11 and J-009349-12) was obtained from DNA technology and ligated into a p3xFLAG-CMV14 vector (Sigma-Aldrich). To generate MTHFD2 Q132K:D155A, GeneArt Site-Directed Mutagenesis PLUS kit was used on the siRNA-resistant MTHFD2 cDNA to introduce point mutations. All sequences were verified by sequencing (KI gene).

### Generation of stable MTHFD2-overexpressing cells

Competent TOP10 *Escherichia coli* cells were transformed by heat shock using 50 ng of plasmid DNA, allowed to recover in SOC (super optimal broth with catabolite repression) medium, then plated on ampicillin agar plates and incubated at 37 °C overnight. Single colonies were expanded in Luria–Bertani (LB) medium incubated on a shaker at 37 °C and 220 r.p.m. overnight. Plasmid DNA was extracted using the PureYield Plasmid Miniprep System (Promega), and DNA concentration was determined using a NanoDrop 8000 spectrophotometer (Thermo Fisher Scientific). Transfection was performed on log(phase) U2OS cells using 4 µg of plasmid DNA and jetPEI transfection reagent (Polyplus-Transfection), according to the manufacturer’s protocol. Successful and stable plasmid integration was maintained by culturing the cells in complete medium supplemented with 250 μg ml^−1^ of G418 disulfate (Sigma-Aldrich).

### Transient siRNA knockdown of MTHFD2

Depletion of endogenous MTHFD2 was achieved using three different siRNA oligonucleotides targeting MTHFD2 (Dharmacon). Nontargeting siRNA was used as a control (Dharmacon). Transfection was performed on log(phase) cells using 10 nM siRNA and INTERFERin transfection reagent (Polyplus-Transfection), following the manufacturer’s protocol. Briefly, cells were seeded 1 d before transfection and allowed to attach overnight. Control and targeting siRNAs were mixed with INTERFERin in serum-free medium, vortexed for 10 s and incubated at room temperature for 15 min to allow transfection complex formation. Cells were washed and changed to fresh complete medium before adding the transfection mix. The cells were incubated with the siRNAs at 37 °C with 5% CO_2_ in a humidified incubator until harvesting.

### Western blotting

Cells were washed and resuspended in 40 µl of lysis buffer: 100 mM Tris-HCl, pH 8, 150 mM NaCl, 1% NP-40, cOmplete protease inhibitor cocktail (Roche) and Halt phosphatase inhibitor cocktail (Thermo Fisher Scientific). Samples were incubated on ice for 30 min, sonicated 3× for 10 s at 100% amplitude, then centrifuged at maximum speed for 15 min. Cleared lysates were used for protein quantification using the Pierce BCA Protein Assay Kit (Thermo Fisher Scientific). Per sample, 20 µg of protein was denatured and reduced with NuPage LDS sample buffer and NuPage sample reducing agent (Thermo Fisher Scientific) at 70 °C for 10 min, then resolved on a 4–15% PROTEAN TGX precast gel (BioRad) at 120 V for approximately 1 h. The separated proteins were transferred on to nitrocellulose membranes (BioRad), followed by blocking with Odyssey Blocking Buffer in Tris-buffered saline (TBS; LI-COR Biosciences). Membranes were incubated in primary antibody solutions (MTHFD2 1:500, p-ATR Ser428 1:500, p-Chk1 Ser345 1:250, RPA32 1:500, γH2AX 1:1,000, cleaved caspase-3 1:1,000, cleaved PARP 1:500, Mcm6 1:500, PCNA 1:500, DNA Pol δ 1:500, RPA70 1:500, RPA32 1:500) at 4 °C overnight. Actin or histone H3 served as a loading control (1:10,000; Abcam). After 3× 5-min washes in 0.1% TBS–Tween 20, the membranes were incubated with IRDye 800CW secondary antibodies (1:5,000; LI-COR Biosciences) at room temperature for 1 h. Fluorescent antibody signal was visualized using an Odyssey Fc imager (LI-COR Biosciences) and quantified using ImageJ software.

### Flow cytometry cell-cycle analysis

Cells were collected, washed and resuspended in 300 µl of phosphate-buffered saline (PBS), then fixed by adding 700 µl of ice-cold absolute ethanol dropwise while vortexing. Samples were incubated on ice for 30 min, then kept at 4 °C at least overnight. After fixation, cells were washed twice with 2% bovine serum albumin (BSA) in PBS and resuspended in 500 µl of propidium iodide (PI) staining solution: 40 µg ml^−1^ of PI, 100 µg ml^−1^ of RNase A and 0.1% Triton X-100 in PBS. Cells were incubated at room temperature for 20 min then analyzed for DNA content using a Navios flow cytometer (Beckman Coulter). At least 10,000 events were acquired per sample and collected data were processed using Kaluza software v.1.3 (Beckman Coulter).

### DNA fiber assay

As previously described^29^, nascent DNA was labeled after 24 h of drug treatment by sequentially changing cell medium to prewarmed medium containing 25 µM of 5-chloro-2′-deoxyuridine (CldU; Sigma-Aldrich, C6891) followed by 250 µM of 5-iodo-2′-deoxyuridine (IdU; Sigma-Aldrich, 57830) for 30 min each. Cells were washed and resuspended to equal density in PBS. Per sample, 10 µl of cell suspension was lysed and spread in a multi-channel μ-slide (Ibidi) using 30 µl of spreading buffer (200 mM Tris-HCl, pH 7.4, 50 mM EDTA, 0.5% sodium dodecylsulfate) and air dried for 30 min. After overnight fixation at 4 °C with methanol/acetic acid (3:1), DNA fibers were denatured with 2.5 M HCl and blocked with 2% BSA/0.1% Tween 20/PBS at room temperature for 1 h each. Fibers were stained with rat anti-bromodeoxyuridine (BrdU) antibody (which detects CldU but not IdU, catalog no. MCA2060, 1:1,000, BioRad) and mouse anti-BrdU antibody (which detects IdU but not CldU, catalog no. 347580, 1:1,000, BD Biosciences) at 37 °C for 1 h, then probed with goat anti-rat and donkey anti-mouse Alexa-Fluor secondary antibodies (1:500, Thermo Fisher Scientific) at room temperature for 2.5 h. Fluorescently labeled fibers were preserved using ProLong Gold mountant (catalog no. P36934, Invitrogen) and images acquired using a LSM-780 confocal microscope (Zeiss) with a ×63 oil objective. Red- and green-track lengths for at least 200 fibers per sample were measured using ImageJ software.

### Confocal imaging

U2OS cells were grown on sterilized glass coverslips, subjected to treatment and fixed in 4% paraformaldehyde and 2% sucrose in PBS for 15 min at room temperature. The cells were permeabilized using 0.2% NP-40 in PBS for 10 min at room temperature, followed by incubation with blocking buffer (2% BSA/0.1% Tween 20/PBS). Coverslips were incubated in a humidified chamber with primary antibody dilution in blocking buffer overnight at 4 °C, rinsed and incubated with secondary antibody dilution and DAPI in blocking buffer for 1 h at room temperature away from light. The coverslips were rinsed, mounted with ProLong Gold mounting medium (catalog no. P36934, Invitrogen) and allowed to cure for 24 h at 4 °C on a flat surface away from light. Images were acquired with a Zeiss LSM 780 confocal microscope using a ×40 or ×63 immersion oil objective. For high-throughput imaging, cells were fixed, using the same protocol as above, in 96-well plates (BD Falcon), and images were taken with an Image Xpress (Molecular Devices) high-throughput microscope. Image analysis was performed using ImageJ and CellProfiler software.

### Colony formation assay

Log(phase) U2OS cells were collected by trypsinization, counted using 0.4% Trypan Blue solution (BioRad) and a TC20 Automated Cell Counter (BioRad), then seeded into 6-well plates at 200 cells per well and allowed to attach overnight. The following day, cells were treated with test compounds as indicated in each experiment and kept in culture for 7–10 d. Colonies were gently washed in PBS, stained with 4% methylene blue in methanol for 30 min, and counted manually.

### Annexin V apoptosis assay

Cells were collected together with culture medium, centrifuged and washed with ice-cold PBS. Per sample, 250,000–500,000 cells were collected and resuspended in 100 µl of 1× binding buffer (10 mM Hepes–NaOH pH 7.4, 140 mM NaCl, 2.5 mM CaCl_2_), then stained at room temperature for 15 min using the FITC-Annexin V Apoptosis Detection Kit (BD Biosciences). After incubation with FITC-Annexin V and PI, 400 µl of 1× binding buffer was added to the samples and immediately analyzed by flow cytometry using a Navios flow cytometer (Beckman Coulter). At least 20,000 events were acquired per sample, and collected data were processed using Kaluza software v.1.3 (Beckman Coulter). Two-way analysis of variance (ANOVA) with Tukey’s correction for multiple comparisons was used to determine significant differences in FITC- and PI-positive populations between treatments and across timepoints. All statistical analyses were performed using Prism v.8.0 (GraphPad Software).

### Resazurin cell viability assay

Test compounds were dissolved in dimethylsulfoxide (DMSO) to a stock of 10 mM and dispensed to their final concentrations in 384-well microplates (Corning) using Echo 550 Liquid handler (Labcyte) or 300e Digital Dispenser (Tecan) for synergy studies. Cells were seeded at a density of 500–2,000 cells per well in 50 µl of medium supplemented with 5% FBS and penicillin/streptomycin, and incubated for 96 h at 37 °C and 5% CO_2_. Cell viability was determined by adding resazurin to a final concentration of 10 µg ml^−1^ (Sigma-Aldrich) and measuring conversion to resorufin after 4–6 h. Fluorescence at 595 nm was measured using a Hidex Sense plate reader, and half-maximal effective concentration (EC_50_) values were calculated using a logistic nonlinear regression model in Prism v.8.0 (GraphPad Software) or parameter logistic model, or sigmoidal dose–response model in XLfit 5 (IDBS Software). Normalized viability data were used to calculate drug combination synergy scores using SynergyFinder^59^. Synergy scores were calculated using the zero-interaction potency reference model and baseline correction.

### Modified comet assay with UNG

The comet assay was performed as previously described^60^. Detailed protocol can be found in Supplementary Methods^61^.

### DARTS

As previously described^62^, HL-60 cells were maintained in a logarithmic growth phase and treated in culture with DMSO (0.01%, v:v) or TH9619 at 1 µM or 50 nM for 4 h. Cells were washed twice with PBS and lysed for 10 min in mammalian protein extraction lysis buffer M-PER (Thermo Fisher Scientific) supplemented with 1× cOmplete protease inhibitor cocktail (Roche). Samples were centrifuged at 16,000*g* and 4 °C for 10 min to clear the lysate from cellular debris. Protein concentration of the cell lysate was determined using the Bradford method with Pierce Coomassie Plus Assay Reagent (Thermo Fisher Scientific). The lysates were diluted in 1× TN buffer (50 mM Tris-HCl, pH 8.0, 50 mM NaCl) and divided into 20-µg aliquots, which were digested with pronase (Roche) at the indicated pronase:protein ratios for 30 min at room temperature. Only 1× TN buffer was added to the nondigested sample instead of protease. Digestion reactions were stopped by adding 4× Laemmli sample buffer (BioRad) supplemented with 100 mM dithiothreitol and heating at 95 °C for 5 min. Proteins were resolved by sodium dodecylsulfate–polyacrylamide gel electrophoresis and protein stabilization was analyzed on western blots according to standard procedures (α-MTHFD2 1:500, α-TYMS 1:1,000, α-SHMT2 1:500, α-DHFR 1:500, α-MTHFD1L 1:500). Superoxide dismutase 1 was used as a loading control (1:1,000; Santa Cruz Biotechnology).

### CETSA

For CETSA^63^ experiments with intact cells treated in culture, cells were seeded at a density of 350,000 cells ml^−1^ and treated with DMSO (0.01%, v:v) or 1 µM test compound for 2 h at 37 °C and 5% CO_2_ in a humidified incubator. Cells were washed once in 1× TBS (50 mM Tris-HCl, pH 7.6, 150 nM NaCl), resuspended in 1× TBS supplemented with protease inhibitors (Roche), then divided into 30-µl aliquots corresponding to approximately 600,000 cells per sample in PCR strip tubes. Cells were heated in a Veriti Thermal Cycler (Applied Biosystems) for 3 min and cooled for 5 min at room temperature to allow precipitation of aggregated proteins. Cell lysis was performed by freeze–thaw cycle 3× at −80 °C for 5 min and at 37 °C for 5 min. Cell lysates were then centrifuged at 20,000*g* and 4 °C for 20 min to remove cellular debris and insoluble proteins. Supernatant was prepared for western blot analysis according to standard procedures (α-MTHFD2 1:500, α-SHMT2 1:500, α-TYMS 1:1,000, α-MTHFD1L 1:500, α-DHFR 1:500). For isothermal dose–response fingerprint CETSA (ITDRF_CETSA_)^63^, 750,000 cells per sample were seeded in a 96-well microplate and inhibitors were added to cells at serial dilutions from 30 µM to 0.00017 µM (threefold steps). Cells were incubated with inhibitors for 2 h at 37 °C and 5% CO_2_ in a humidified incubator. Cell harvest, heating and lysis procedures were performed identically to those for CETSA experiments with intact cells. For ex vivo CETSA, tumors extracted from mice were grinded and approximately 10 mg of tumor powder from each animal was resuspended in 650 µl of lysis buffer (20 mM TBS, pH 7.6, 0.1% Tween 20 and protease inhibitor cocktail (Roche)). Samples were vortexed and cell suspensions were divided into 50-µl aliquots in PCR strips. Heating of samples and lysis procedures were performed identically to those for CETSA experiments with intact cells. For each condition, tumor samples from four animals were analyzed.

### Physicochemical property calculations

The cLogP and topological polar surface areas were calculated using DataWarrior 5.0.0 (Idorsia Pharmaceuticals Ltd.).

### MTHFD2 high-throughput screening

The assay was run in ProxiPlate low-volume, 384-well plates (Perkin Elmer, catalog no. 6008280). Then, 10,500 lead-like structurally diverse compounds, provided by the Laboratory for Chemical Biology at the Karolinska Institute (LCBKI), were screened at 10 µM. LY345899, 10 µM, was used as a positive control and DMSO as a negative control. The average Z′ (measure of statistical effect size and assay quality) for all plates was 0.82 and the hit threshold was set to the mean inhibition of the samples + 3 s.d.s (68% inhibition). Hits were counter screened at 100 µM but in the absence of MTHFD2. In a similar fashion, approximately 43,000 lead-like compounds selected from Enamine and Maybridge libraries provided by LCBKI and approximately 443,000 lead-like compounds provided by the European Lead Factory^64^ were screened in 1,536-well formats (Supplementary Table 1). Additional protocol details can be found in Supplementary Methods^61^.

### Interaction analysis using surface plasmon resonance biosensor

The experiments were performed using Biacore T200 instrument (Cytiva) at 25 °C. The immobilization of MTHFD2 was carried out by a standard amine coupling procedure on a CM5 biosensor chip (GE Healthcare). MTHFD2 was diluted to 25 µg ml^−1^ in maleic acid buffer, pH 6.2. The CM5 chip surface was activated by an injection of a 1:1 mixture of *N*-ethyl-*N*′-(3-(dimethylamino)propyl)carbodiimide and *N*-hydroxysuccinimide for 7 min, at a flow rate of 10 µl min^−1^. MTHFD2 was injected over the activated surface at a flow rate of 2 µl min^−1^ until the immobilization level reached around 3,000 RU (relative units). Then, the surface was deactivated by the injection of 1 M ethanolamine for 7 min. After immobilization, the concentration series of TH9619 and TH7299, ranging from 7.8 nM to 1,000 nM, was injected over the surface, at a flow rate of 90 µl min^−1^. The compounds were tested in 1× HBS-P, pH 7.4 (catalog no. BR-1006-70, Cytiva), 1 mM dithiothreitol, 250 µM NAD and 3% DMSO. An association phase was monitored for 60 s and a dissociation phase for 360 s. A solvent correction accounting for 3% DMSO was performed. The data were analyzed using Biacore T200 Evaluation Software, v.3.1 (Cytiva). Sensorgrams were double-referenced by subtracting the signals from a reference surface and the average signals from two blank injections and fitted to a 1:1 Langmuir binding model.

### Synthesis of MTHFD2 inhibitors

All reagents were of commercial grade and used without further purification. Flash column chromatography was performed on Merck silica gel 60 (70–230 mesh). Preparative high-performance liquid chromatography (HPLC) was performed on a Gilson HPLC system: Column ACE 5 C8 (150 × 30 mm^2^); H_2_O (containing 0.1% trifluoroacetic acid (TFA)) and MeCN were used as the mobile phases. Analytical LC–MS was conducted using an Agilent MSD mass spectrometer connected to an Agilent 1100 HPLC. System A: Column ACE 3 C8 (50 × 3.0 mm^2^); H_2_O (0.1% TFA) and MeCN were used as the mobile phases; system B: Xterra MS C18 (50 × 3.0 mm^2^); H_2_O (containing 10 mM NH_4_HCO_3_, pH 10) and MeCN were used as the mobile phases. All compounds gave satisfactory purities when analyzed using both systems. Nuclear magetic resonance spectra of ^1^H were recorded on a Bruker Advance DPX 400 spectrometer at 400.1 MHz. High-resolution mass spectra were collected on a maXis Impact TOF mass spectrometer. Synthesis of TH7299 (ref. ^65^) and DS18561882 (ref. ^24^) was performed as described. Synthesis and analysis of TH9028, TH9619 and TH11737 (the enantiomer of TH9619) are detailed in Supplementary Methods^61^.

### Genomic dU measurements

DNA from MTHFD2 inhibitor-treated cells was isolated for nucleoside quantification by phenol:chloroform:isoamyl alcohol extraction as previously described^38,66^. Protocol details can be found in Supplementary Methods^61^.

### Cocrystallization

Inhibitor (TH7299, TH9028 or TH9619) 5 mM, 5 mM NAD^+^ and 6 mM MgCl_2_ were added to MTHFD2 protein and incubated for 20 min. Then, 10 mM of Na_2_HPO_4_ was added, after which the sample was incubated for an additional 10 min. Proteases (1:50 ratio each of trypsin, α-chymotrypsin, pepsin, papain, proteinase K and subtilisin) were added immediately before crystallization. For crystallization at 20 °C, MTHFD2 at 9.66 mg ml^−1^ was mixed in a 3:1 ratio with either 0.1 M phosphate/citrate, pH 4.3, 42% (v:v) PEG300 (MTHFD2–TH7299), 0.1 M Hepes, pH 7.8, 80% (v:v) 2-methyl-2,4-pentanediol (MTHFD2–TH9028) or 0.1 M Hepes, pH 7.6, 75% (v:v) MPD (MTHFD2–TH9619). After 1 week, crystals were frozen in liquid nitrogen.

### Crystallography data collection, structure determination and refinement

Datasets were collected for MTHFD2–TH7299 and MTHFD2–TH9619 at stations I03 and I04 of the Diamond Light Source equipped with a PILATUS 6M detector. Data for MTHFD2–TH9028 were collected at beamline PXI of the Swiss Light Source equipped with an EIGER 16M detector. Complete datasets were collected on single crystals at 100,000 for each complex. Datasets were processed and scaled with XDS^67^, xia2 (ref. ^68^), DIALS^69^ and AIMLESS^70^ within the CCP4 suite^71^. The structures were solved via molecular replacement with Phaser^72^ using a previously solved MTHFD2 structure (PDB accession no. 5TC4) as the search model. Several cycles of manual model building and refinement were performed using Coot^73^ and Refmac5 (ref. ^74^), during which waters and ligands were added to the structure. Data collection and refinement statistics are shown in Supplementary Table 3. The coordinates and structure factors for MTHFD2–TH7299, MTHFD2–TH9028 and MTHFD2–TH9619 were deposited in the Protein Data Bank (PDB) under accession nos. 6S4E, 6S4A and 6S4F, respectively.

### Differential scanning fluorimetry-based selectivity screening against a curated kinase library

The assay was performed as previously described^75^. Briefly, recombinant protein kinase domains at a concentration of 2 μM in 10 mM Hepes, pH 7.5, and 500 mM NaCl were mixed with 12 μM TH9619. Temperature-dependent protein unfolding profiles were measured using a real-time PCR Mx3005p machine (Stratagene). Staurosporine was used as a positive control. Experiments were performed in triplicate.

### Eurofins SafetyScreen44 panel

A list of 44 recommended targets to provide an early identification of significant off-target interactions and potential hazards of compounds has previously been curated^76^. Reference standards were run as part of each assay. Biochemical assay results are presented as the percentage inhibition of specific binding or activity. For all targets, the significance criteria were set at ≥ 50% of maximal stimulation or inhibition. Experiments were performed in duplicate.

### In vivo mouse studies

Approximately 6- to 8-week-old female mice were used in all our efficacy studies. Mice were housed four per cage in individually ventilated cages (type IVC) in a 12:12 light:dark cycle. Room temperature/ambient was kept at 21 ˚C ± 4˚C and humidity 40–70%. Animals were fed a folic acid-deficient diet (ENVIGO, Teklad customized diet, catalog no. TD.01013) or rodent maintenance pelleted diet (SDS, catalog no. 801151). Food and water were provided freely. For the pharmacokinetic evaluation of TH9619, NOD.Cg-Prkdcscid Il2rgtm1Sug/JicTac (NOG) mice (Taconic Biosciences) were used. The repeated dose tolerability study of TH9619 was performed on NOD.CB17-Prkdcscid/NCrCrl (NOD–SCID) mice (Charles River Laboratories). For the HL-60 tumor xenograft models, NOG mice were used in the TH9619 efficacy study on standard chow versus LF, whereas the survival study comparing TH9619 with AraC was performed on NOD.Cg-*Prkdc*^*scid*^ *Il2rg*^*tm1Wjl*^/SzJ (NSG) mice (Jackson Laboratory). In the xenograft studies, mice were kept on an LF for the duration of the studies, starting 2 weeks before cell injections. Per mouse, 5 million HL-60 cells were injected via the tail vein into 7-week-old NOG or NSG mice. The leukemia burden was assessed by palpation. Body weight was monitored regularly and blood was collected at the time of sacrifice to run hematology and clinical chemistry panels, as well as measure thymidine and 5-MTHF levels in all animals, including satellite animals. Tumors were excised after sacrifice and used for target engagement analysis. All experiments in the present study were performed in accordance with the guidelines from the Swedish National Board for Laboratory Animals and the European Community Council Directive (86/609/EEC), and approved by the Swedish Ethical Committee (ethical permits N217/15 and N89/14). The maximal tumor size permitted by the Swedish Ethical Committee is 1,000 mm^3^, a limit that was not exceeded in our experiments.

### Statistics and reproducibility

No statistical method was used to predetermine sample size but our sample sizes are similar to those reported in previous publications^19,29^. No data were excluded from the analyses. The experiments were not randomized, and the investigators were not blinded to allocation during experiments and outcome assessment. Wherever statistics have been derived, exact *n* values have been listed in the figure legends and replicates explicitly defined. Raw numerical data and statistical analysis of all repeats for all figures and extended data are provided in Source Data.

### Reporting Summary

Further information on research design is available in the Nature Research Reporting Summary linked to this article.

## Supplementary information

Supplementary Information Supplementary Fig. 1.

Reporting Summary.

Supplementary Table 1 Supplementary Tables 1–5.

## Data Availability

All data generated or analyzed during the present study, including source data, can be found in the article, Extended Data or Supplementary Information. The coordinates and structure factors for MTHFD2–TH7299, MTHFD2–TH9028 and MTHFD2–TH9619 cocrystal structures were deposited in the PDB under accession nos. 6S4E, 6S4A and 6S4F, respectively. Additional datasets generated during the present study and relevant information are available from the corresponding authors upon request. Source data are provided with this paper.

## Source data

Source Data Fig. 1 Statistical source data.
Source Data Fig. 2 Statistical source data.
Source Data Fig. 2 Unprocessed western blots.
Source Data Fig. 3 Statistical source data.
Source Data Fig. 4 Statistical source data.
Source Data Fig. 4 Unprocessed western blots.
Source Data Fig. 5 Statistical source data.
Source Data Fig. 6 Statistical source data.
Source Data Fig. 6 Unprocessed western blots.
Source Data Extended Data Fig. 1 Statistical source data.
Source Data Extended Data Fig. 2 Statistical source data.
Source Data Extended Data Fig. 2 Unprocessed western blots.
Source Data Extended Data Fig. 4 Statistical source data.
Source Data Extended Data Fig. 4 Unprocessed western blots.
Source Data Extended Data Fig. 5 Statistical source data.
Source Data Extended Data Fig. 6 Statistical source data.
Source Data Extended Data Fig. 7 Statistical source data.
Source Data Extended Data Fig. 8 Statistical source data.
Source Data Extended Data Fig. 9 Statistical source data.
Source Data Extended Data Fig. 10 Statistical source data.
Source Data Extended Data Fig. 10 Unprocessed western blots.

## Extended data

Extended data are available for this paper at 10.1038/s43018-022-00331-y.
