# Supplementary material for: Pharmacological targeting of MTHFD2 suppresses acute myeloid leukemia by inducing thymidine depletion and replication stress
Source: Nat Cancer. 2022 Feb 28;3(2):156–72. doi: 10.1038/s43018-022-00331-y (PMC8885417; doi:10.1038/s43018-022-00331-y)
Supplement: Supplementary file 1 — Supplementary Fig. 1. [file 43018_2022_331_MOESM1_ESM.pdf]

nature portfolio

https://doi.org/10.1038/s43018-022-00331-y

Supplementary information

Pharmacological targeting of MTHFD2 suppresses acute myeloid leukemia by inducing thymidine depletion and replication stress

In the format provided by the authors and unedited

Pharmacological targeting of MTHFD2 suppresses acute myeloid leukemia by inducing thymidine depletion and replication stress

Bonagas *et al*.

Supplementary Information

- Supplementary Figure 1. Flow cytometry gating strategy

Flow cytometry gating strategy examples

EdU-γH2AX-Hoechst (DNA damage and cell cycle assay)

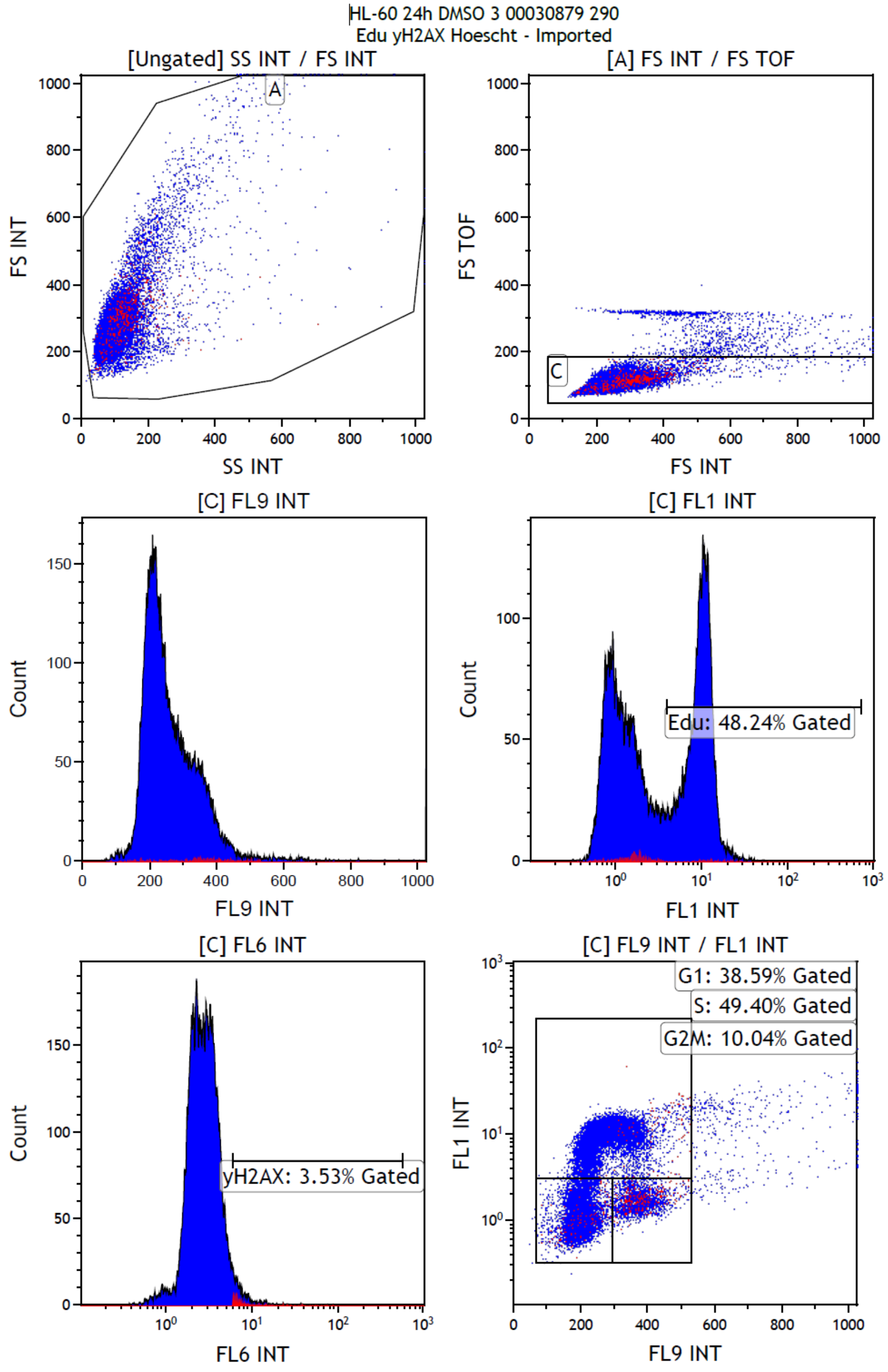

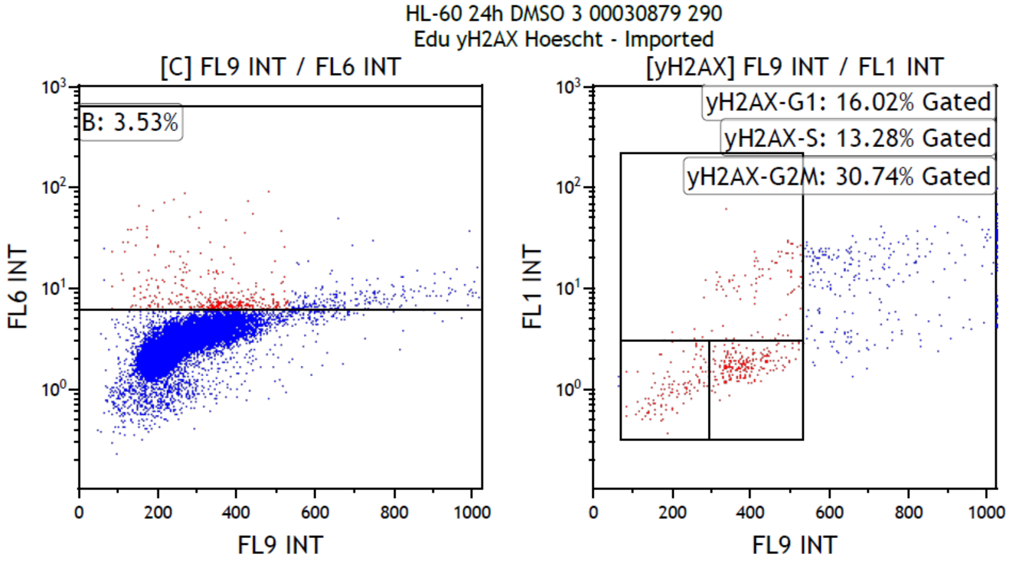

Gate	Number	%Total	%Gated	Logic
C	19,631	94.16	94.33	C AND A
B	693	3.32	3.53	B AND C AND A
Edu	9,470	45.42	48.24	Edu AND C AND A
G1	7,575	36.33	38.59	G1 AND C AND A
G2M	1,970	9.45	10.04	G2M AND C AND A
S	9,698	46.52	49.40	S AND C AND A
yH2AX	693	3.32	3.53	yH2AX AND C AND A
yH2AX-G1	111	0.53	16.02	yH2AX-G1 AND yH2AX AND C AND A
yH2AX-G2M	213	1.02	30.74	yH2AX-G2M AND yH2AX AND C AND A
yH2AX-S	92	0.44	13.28	yH2AX-S AND yH2AX AND C AND A

AnnexinV-FITC and PI (apoptosis assay)

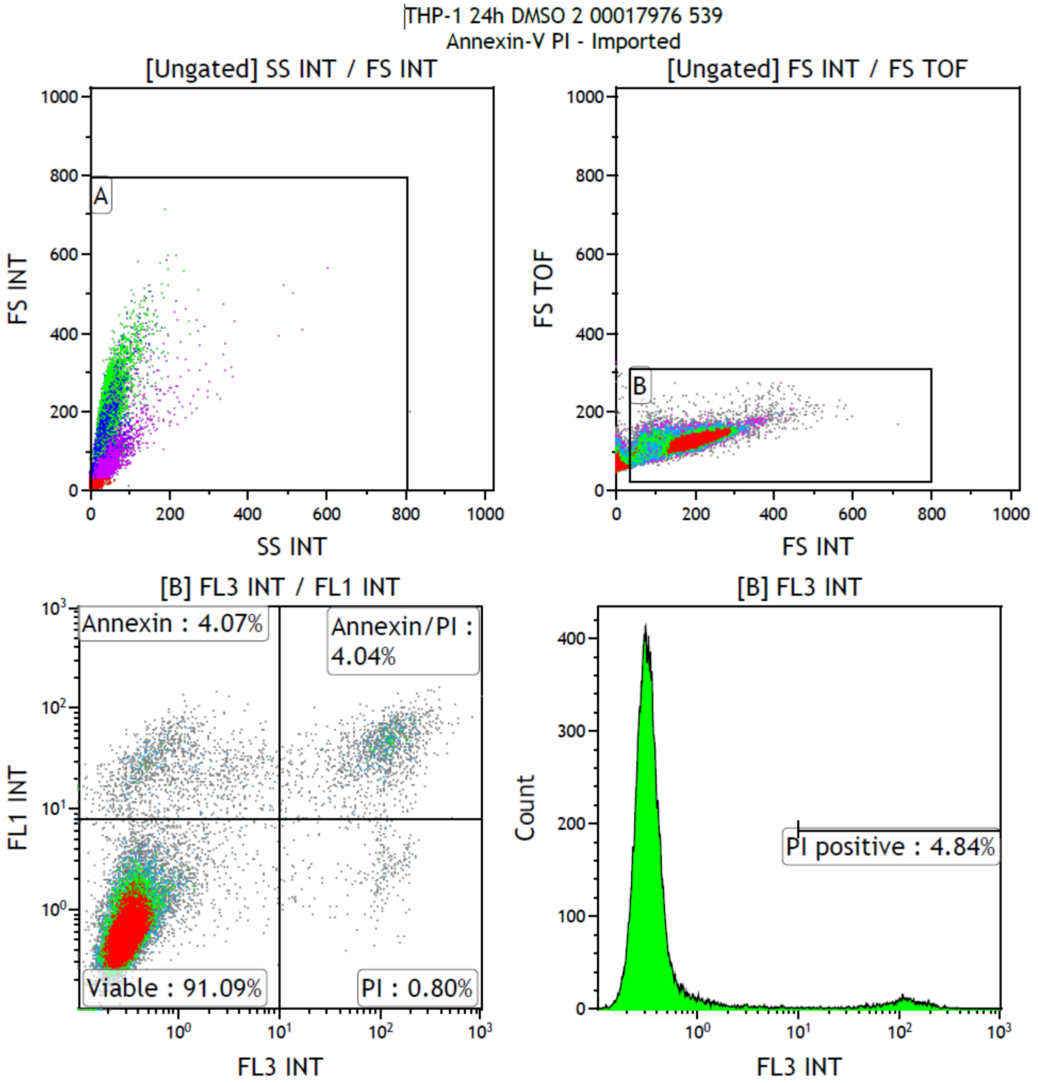

Gate	%Total	%Gated
All	76.19	100.00
Annexin	3.10	4.07
Annexin/PI	3.08	4.04
PI	0.61	0.80
Viable	69.40	91.09

THP-1 24h DMSO 2 00017976 539
Annexin-V PI - Imported

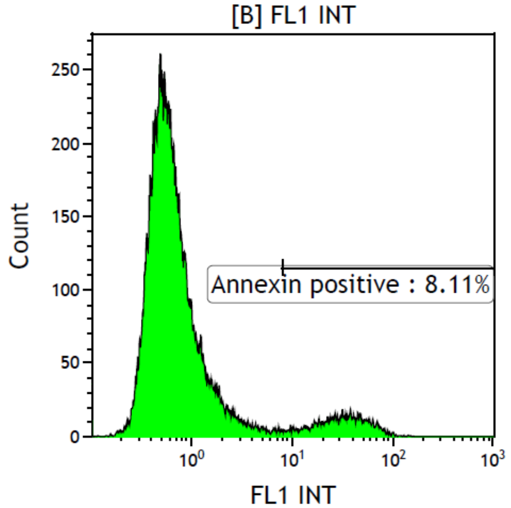

Gate	Number	%Total	%Gated
All	30,475	76.19	100.00
Annexin positive	2,472	6.18	8.11

CD11b-FITC (differentiation assay)

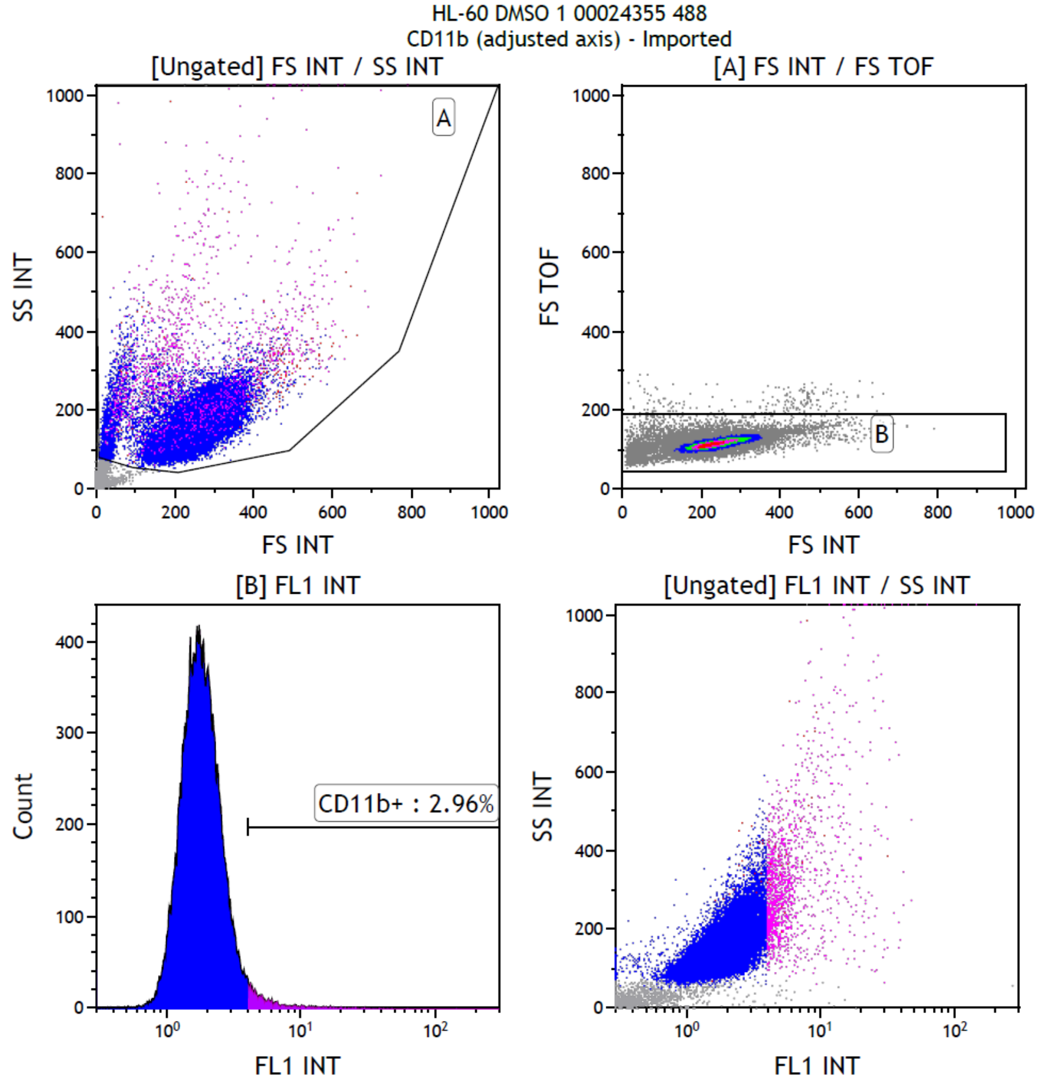

Gate	Number	%Total	%Gated	Logic
All	63,016	100.00	100.00	Ungated
A	49,456	78.48	78.48	A
B	49,250	78.15	99.58	B AND A
CD11b+	1,460	2.32	2.96	CD11b+ AND B AND A

CD11b-FITC and PI (differentiation and cell death assay)

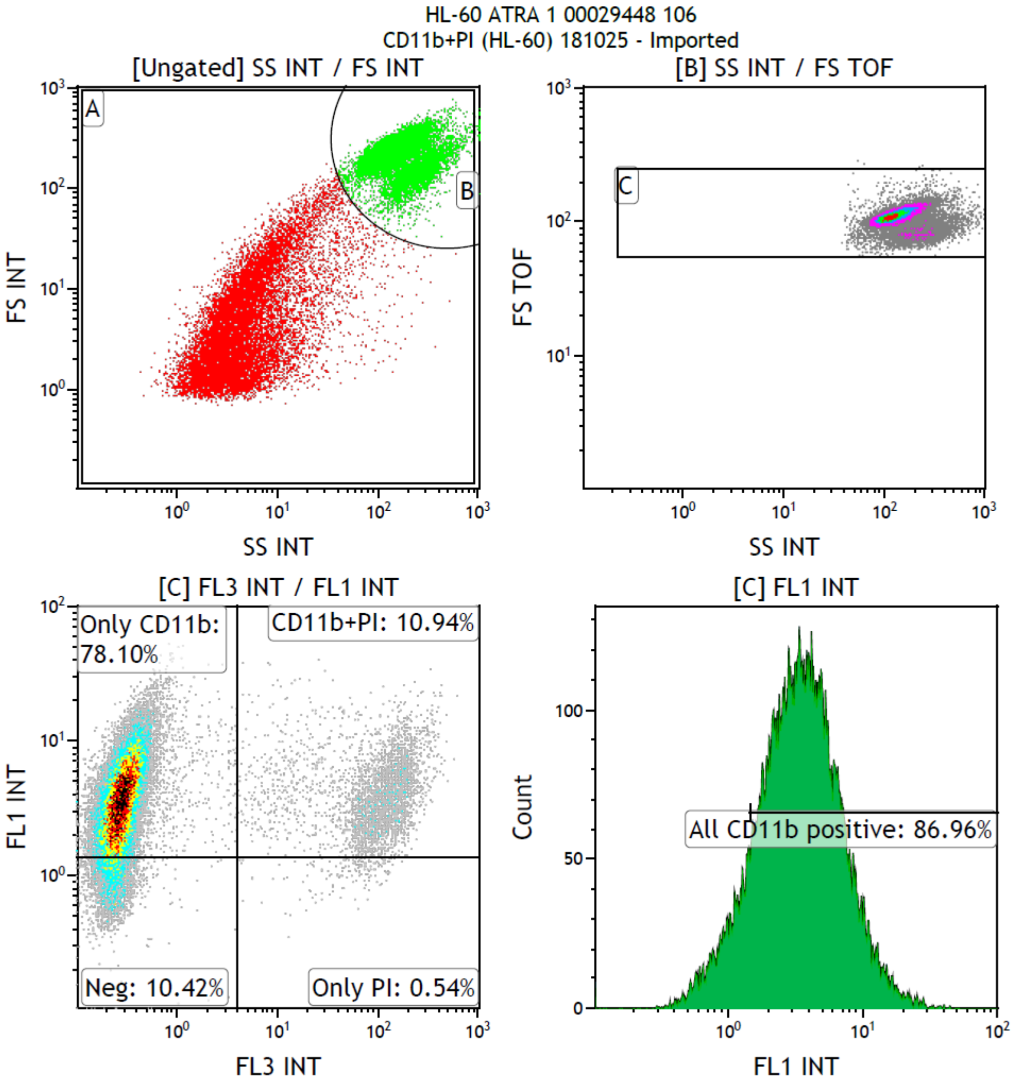

Gate	%Total	%Gated
All	67.95	100.00
CD11b+PI	7.43	10.94
Neg	7.08	10.42
Only CD11b	53.07	78.10
Only PI	0.37	0.54

Gate	Number	%Total	%Gated
All	30,013	67.95	100.00
All CD11b positive	26,098	59.09	86.96

HL-60 ATRA 1 00029448 106
CD11b+PI (HL-60) 181025 - Imported

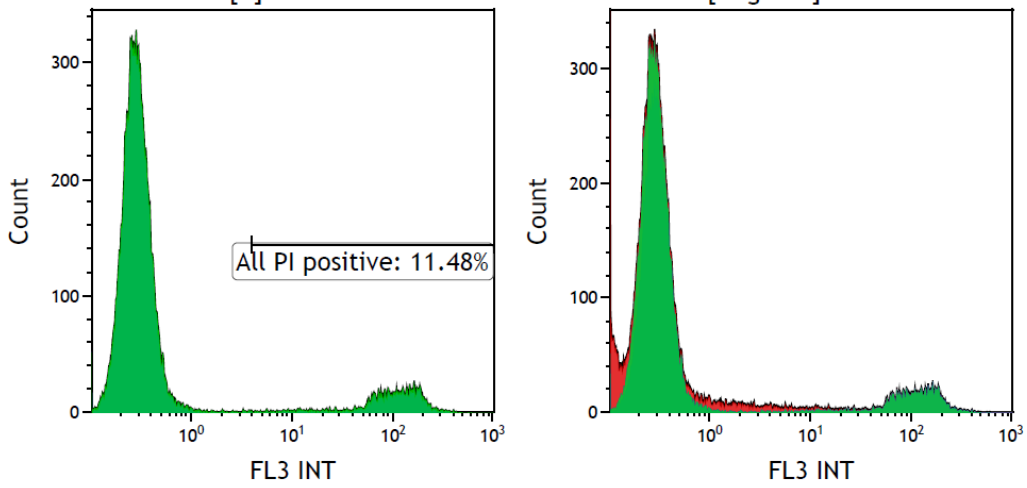

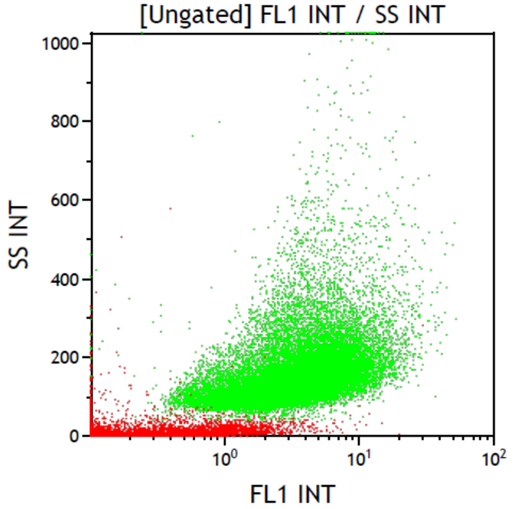

Gate	Number	%Total	%Gated	Logic
All	44,169	100.00	100.00	Ungated
A	44,127	99.90	99.90	A
B	30,019	67.96	67.96	B
C	30,013	67.95	99.98	C AND B
All CD11b positive	26,098	59.09	86.96	"All CD11b positive" AND C AND B
All PI positive	3,445	7.80	11.48	"All PI positive" AND C AND B
CD11b+PI	3,282	7.43	10.94	CD11b+PI AND C AND B
Neg	3,128	7.08	10.42	Neg AND C AND B
Only CD11b	23,440	53.07	78.10	"Only CD11b" AND C AND B
Only PI	163	0.37	0.54	"Only PI" AND C AND B

Supplementary Figure 1. Flow cytometry gating strategy.

In general, the gating strategy used in this study can be summarized as follows: (1) forward and side scatter gating to discard debris and select cells based on size and complexity, (2) time-of-flight (TOF) gating to remove doublets and other cell aggregates, (3) subset gating based on fluorescence markers (single, double or triple staining), with fluorescence-minus-one (FMO), unstained, positive and negative controls used to set cytometer parameters and define the populations of interest for each individual experiment, and (4) color-coded back-gating to visualize cells in the different end-gates from a high level perspective. For all flow cytometry experiments, flow rate was monitored and maintained at approximately 100 events/sec. Events were collected until at least 10,000 individual events were gated in (1) and (2) for each sample, which were then further used for subset gating (3) and statistical analysis of distinct cell populations. An example is given above for each type of flow cytometry experiment performed in this study.
