# Supplementary material for: Pharmacological targeting of MTHFD2 suppresses acute myeloid leukemia by inducing thymidine depletion and replication stress
Source: Nat Cancer. 2022 Feb 28;3(2):156–72. doi: 10.1038/s43018-022-00331-y (PMC8885417; doi:10.1038/s43018-022-00331-y)
Supplement: Supplementary file 2 — Reporting Summary. [file 43018_2022_331_MOESM2_ESM.pdf]

nature research

Corresponding author(s): Thomas Helleday

Last updated by author(s): Nov 29, 2021

Reporting Summary

Nature Research wishes to improve the reproducibility of the work that we publish. This form provides structure for consistency and transparency in reporting. For further information on Nature Research policies, see our Editorial Policies and the Editorial Policy Checklist.

Statistics

For all statistical analyses, confirm that the following items are present in the figure legend, table legend, main text, or Methods section.

n/a	Confirmed	
☐	☒	The exact sample size (*n*) for each experimental group/condition, given as a discrete number and unit of measurement
☐	☒	A statement on whether measurements were taken from distinct samples or whether the same sample was measured repeatedly
☐	☒	The statistical test(s) used AND whether they are one- or two-sided<br>*Only common tests should be described solely by name; describe more complex techniques in the Methods section.*
☒	☐	A description of all covariates tested
☐	☒	A description of any assumptions or corrections, such as tests of normality and adjustment for multiple comparisons
☐	☒	A full description of the statistical parameters including central tendency (e.g. means) or other basic estimates (e.g. regression coefficient) AND variation (e.g. standard deviation) or associated estimates of uncertainty (e.g. confidence intervals)
☐	☒	For null hypothesis testing, the test statistic (e.g. *F*, *t*, *r*) with confidence intervals, effect sizes, degrees of freedom and *P* value noted<br>*Give P values as exact values whenever suitable.*
☒	☐	For Bayesian analysis, information on the choice of priors and Markov chain Monte Carlo settings
☒	☐	For hierarchical and complex designs, identification of the appropriate level for tests and full reporting of outcomes
☒	☐	Estimates of effect sizes (e.g. Cohen's *d*, Pearson's *r*), indicating how they were calculated

Our web collection on statistics for biologists contains articles on many of the points above.

Software and code

Policy information about availability of computer code

Data collection	Image Studio Software 5.2 (LI-COR Biosciences); ZEN Software 2.1 (ZEISS); Hidex Sense Software 1.0 (Hidex Oy); Comet Assay IV 4.3 (Instem); Navios Software 1.2 (Beckman Coulter); Biacore T200 Software 3.1 (Cytiva); Xcalibur Software 4.3 (ThermoScientific)
Data analysis	ImageJ 1.8 (National Institutes of Health, MD, USA); Image Studio Lite Software 5.2 (LI-COR Biosciences); ZEN Lite Software 2.1 (ZEISS); CellProfiler Image Analysis Software 4.0.5 (Broad Institute, MA, USA); Prism Software 8.0.0 (GraphPad Software); PyMOL 2.1.1 (Schrödinger); Kaluza Analysis Software 1.3 (Beckman Coulter); Comet Assay IV 4.3 (Instem); SynergyFinder 2.0 (Institute for Molecular Medicine Finland); XLfit 5 add-in for Excel (IDBS Software); DataWarrior 5.0.0 (Idorsia Pharmaceuticals Ltd.); Biacore T200 Evaluation Software 3.1 (Cytiva); XDS Software (MPI for Medical Research, Heidelberg, Germany); xia2 Software (Diamond Light Source); DIALS Software (Diamond Light Source); AIMLESS Software (MRC Laboratory of Molecular Biology, Cambridge, UK); Phaser Crystallographic Software 2.1.2 (University of Cambridge, UK); Coot (MRC Laboratory of Molecular Biology, Cambridge, UK); Refmac5 (MRC Laboratory of Molecular Biology, UK); MassHunter Software B.08.00 (Agilent); TraceFinder 4.1 (ThermoScientific)

For manuscripts utilizing custom algorithms or software that are central to the research but not yet described in published literature, software must be made available to editors and reviewers. We strongly encourage code deposition in a community repository (e.g. GitHub). See the Nature Research guidelines for submitting code & software for further information.

Data

Policy information about availability of data

All manuscripts must include a data availability statement. This statement should provide the following information, where applicable:

- Accession codes, unique identifiers, or web links for publicly available datasets
- A list of figures that have associated raw data
- A description of any restrictions on data availability

All data generated or analyzed during this study, including source data, can be found in the article or in the Supplementary Information. The coordinates and structure factors for MTHFD2-TH7299, MTHFD2-TH9028 and MTHFD2-TH9619 co-crystal structures were deposited in the PDB under the accession codes 6S4E, 6S4A and 6S4F, respectively. Additional datasets generated during the current study and relevant information are available from the corresponding authors upon request.

Field-specific reporting

Please select the one below that is the best fit for your research. If you are not sure, read the appropriate sections before making your selection.

☒ Life sciences ☐ Behavioural & social sciences ☐ Ecological, evolutionary & environmental sciences

For a reference copy of the document with all sections, see nature.com/documents/nr-reporting-summary-flat.pdf

Life sciences study design

All studies must disclose on these points even when the disclosure is negative.

Sample size	For imaging experiments, between 100-200 cells or DNA fibers were imaged and quantified per condition, similar to previous publications (Petermann et al., 2010. Hydroxyurea-Stalled Replication Forks Become Progressively Inactivated and Require Two Different RAD51-Mediated Pathways for Restart and Repair. Mol. Cell 37, 492–502). For flow cytometry experiments, between 10,000-30,000 single cell events were acquired and quantified per replicate, per condition, following the guidelines outlined by Cossarizza et al., 2019. Guidelines for the use of flow cytometry and cell sorting in immunological studies (second edition). Eur J Immunol; 49(10):1457-1973. Unless otherwise stated, n=2 was chosen as the minimal number of technical replicates per experiment that would allow for adequate analysis to draw meaningful conclusions of the data. We determined this to be sufficient based on the low observed variability between samples from cell culture in vitro experiments. However, in in vivo studies, higher variability is observed, therefore a larger sample size (n>5) was used to compensate for this natural variance. In vivo study sample size is similar to previous publications (Pikman et al., 2016. Targeting MTHFD2 in acute myeloid leukemia. J. Exp. Med. 213, 1285–1306). No statistical estimation of sample size was performed.
Data exclusions	No data were excluded from analysis
Replication	Except for the in vivo studies, all experiments presented in this study were performed at least twice under independent experimental conditions. All attempts at replication have been successful. To ensure reproducibility, reagent suppliers and catalog numbers were maintained as long as possible. When new reagents needed to be used, validation and optimization experiments were conducted to ensure comparable results.
Randomization	For the in vivo studies, animals were assigned randomly to experimental and control groups. The rest of the experiments were not randomized, but independent replicates were often performed in different formats (e.g., dose-response serial dilutions in 96-well plates vs. 384-well plates, by column or by row, etc.) and by different investigators, as mitigation measures to cancel out experimental bias.
Blinding	Blinding was not possible for any experiment as treatment conditions were evident from the data. Quantifications were performed using computational pipelines (CellProfiler and Comet Assay IV for imaging data, Kaluza for flow cytometry data) applied equally to all conditions and replicates for any given experiment. Thresholds for detecting foci and staining-positive cells were chosen for each experiment based on internal controls but applied equally across all conditions and replicates within a same experiment.

Reporting for specific materials, systems and methods

We require information from authors about some types of materials, experimental systems and methods used in many studies. Here, indicate whether each material, system or method listed is relevant to your study. If you are not sure if a list item applies to your research, read the appropriate section before selecting a response.

Materials & experimental systems

n/a	Involved in the study
☐	☒ Antibodies
☐	☒ Eukaryotic cell lines
☒	☐ Palaeontology and archaeology
☐	☒ Animals and other organisms
☒	☐ Human research participants
☒	☐ Clinical data
☒	☐ Dual use research of concern

Methods

n/a	Involved in the study
☒	☐ ChIP-seq
☐	☒ Flow cytometry
☒	☐ MRI-based neuroimaging

Antibodies

Antibodies used

Anti-β-Actin (Abcam, Cat. # ab6276, 1:10,000); Anti-MTHFD2 (Abcam, Cat. # ab56772, 1:500); Anti-RPA32/RPA2 (4E4) (Cell Signaling Technology, Cat. # 2208, 1:500); Anti-RPA70 (Cell Signaling Technology, Cat. # 2267, 1:500); Anti-phospho-RPA2 (Ser33) (Novus Biologicals, Cat. # NB100-544, 1:500); Anti-cleaved PARP (Asp214) (Cell Signaling Technology, Cat. # 9541, 1:500); Anti-phospho-Chk1 (Ser345) (Cell Signaling Technology, Cat. # 2341, 1:250); Anti-phospho-Cdk (Thr14/Tyr15) (Santa Cruz Biotechnology, Cat. # sc-28435-R, 1:500); Anti-phospho-H2A.X (Ser139) (Cell Signaling Technology, Cat. # 2577, 1:1,000); Anti-phospho-H2A.X (Ser139) (Millipore, Cat. # 05-636, 1:1,000); Anti-cleaved caspase-3 (Asp175) (Cell Signaling Technology, Cat. # 9661, 1:1,000); Anti-p21 (Santa Cruz Biotechnology, Cat. # sc-756, 1:500); Anti-MCM6 (Abcam, Cat. # ab4458, 1:500); Anti-PCNA (Santa Cruz Biotechnology, Cat. # sc-25280, 1:500); Anti-DNA polymerase delta (Abcam, Cat. # ab10362, 1:500); Anti-histone H3 (Abcam, Cat. # ab1791, 1:10,000); Anti-SOD-1 (G-11) (Santa Cruz Biotechnology, Cat. # sc-17767, 1:1,000); Anti-DHFR (Santa Cruz Biotechnology, Cat. # sc-377091, 1:500); Anti-TYMS (Cell Signaling Technology, Cat. # 9045, 1:1,000); Anti-MTHFD1 (Antibodies Online, Cat. # ABIN389285); Anti-MTHFD1L (Abcam, Cat. # ab229708, 1:500); Anti-SHMT1 (Abcam, Cat. # ab55736, 1:500); Anti-SHMT2 (Abcam, Cat. # ab180786, 1:500); Anti-FPGS (Novus, Cat. # NBP2-16526, 1:500); Anti-Mouse IgG IRDye® 800CW (LI-COR Biosciences, Cat. # 926-32212, 1:5,000); Anti-Rabbit IgG IRDye® 800CW (LI-COR Biosciences, Cat. # 926-32213, 1:5,000); Anti-Rat IgG IRDye® 800CW (LI-COR Biosciences, Cat. # 925-32219, 1:5,000); Anti-CD11b FITC Conjugated (Beckman Coulter, Cat. # IM0530U, 1:100); Anti-BrdU/CldU (Bio-Rad / AbD Serotec, Cat # MCA2060, 1:1,000); Anti-BrdU/IdU (BD Biosciences, Cat. # 347580, 1:1,000); Anti-Rat IgG Alexa Fluor® 568 (Thermo Fisher Scientific, Cat. # A-11077, 1:500); Anti-Mouse IgG Alexa Fluor® 488 (Thermo Fisher Scientific, Cat. # A-21202, 1:500)

Validation

All antibodies have been validated for use in their respective applications (immunofluorescence, flow cytometry and/or western blotting), as stated on the manufacturers' product pages.

Anti-β-Actin: KO validated. Suitable for ICC/IF, WB. Reacts with Mouse, Rat, Cow, Dog, Human, African green monkey, Chinese hamster. HeLa, Jurkat, COS-7, NIH/3T3, PC-12, Rat2, CHO, MDBK and MDCK (Dox-inducible β-actin) whole cell lysates were used as positive controls.
Anti-MTHFD2: KO validated. Suitable for WB, IHC-P, Flow Cyt. Reacts with Human. HEK293T, HepG2 and HeLa whole cell lysates were used as positive controls.In-house validation for IF using siRNA KD.
Anti-RPA32/RPA2 (4E4): Suitable for WB, IP, IF/ICC, Flow Cyt. Reacts with Human, Mouse, Rat, Hamster, Monkey.
Anti-RPA70: Suitable for WB, IP, IF/ICC, Flow Cyt. Reacts with Human, Monkey.
Anti-phospho-RPA2 (Ser33): Suitable for WB, ICC/IF. Reacts with Human, Mouse, Orangutan. Biological Strategies Validation (defined biological or chemical modulation of protein expression to demonstrate antibody specificity to the target protein. The data is compared across multiple cell lines including positive and negative expressing cells, and multiple species, if applicable). RPA2 overexpression lysate used as positive control.
Anti-cleaved PARP (Asp214): Suitable for WB. Reacts with Human. Etoposide treatment used as positive control.
Anti-phospho-Chk1 (Ser345): Suitable for WB. Reacts with Human, Mouse, Rat, Monkey. UV and MMS used as positive controls.
Anti-phospho-Cdk (Thr14/Tyr15): Suitable for WB, IP, IF, ELISA. Reacts with Human, Mouse, Rat. Nocodazole used as positive control.
Anti-phospho-H2A.X (Ser139) (Cell Signaling Technology): Suitable for WB, IF/ICC, Flow Cyt. Reacts with Human, Mouse, Rat, Monkey. UV used as positive control.
Anti-phospho-H2A.X (Ser139) (Millipore): Suitable for ICC, IF, WB, ChIP, IHC. Reacts with Vertebrates. UV used as positive control.
Anti-cleaved caspase-3 (Asp175): Suitable for WB, IP, IHC-P, IF/ICC, Flow Cyt. Reacts with Human, Mouse, Rat, Monkey. Staurosporine and cytochrome c used as positive controls.
Anti-p21: Suitable for WB, IP, IF, ELISA. Reacts with Human, Mouse, Rat. C32 + PMA nuclear extracts were used as positive controls.
Anti-MCM6: Suitable for WB, IHC-P. Reacts with Human. RIPA extract from HeLa whole cell lysate was used as a positive control.
Anti-PCNA: Suitable for WB, IP, IF, IHC-P, ELISA. Reacts with Human, Mouse, Rat.
Anti-DNA polymerase delta: Suitable for WB, IP. Reacts with Human, Mouse. HeLa whole cell lysate was used as positive control.
Anti-histone H3: Suitable for ICC, IHC-P, ChIP, IP, WB. Reacts with Mouse, Rat, Human, Saccharomyces cerevisiae, Xenopus laevis, Arabidopsis thaliana, Drosophila melanogaster, Indian muntjac, Schizosaccharomyces pombe. A431, Jurkat and HEK293 whole cell lysates, with and without Human Histone H3 peptide at 1 μg/ml, were used as positive and negative controls respectively.
Anti-SOD-1 (G-11): Suitable for WB, IP, IF, IHC-P, ELISA. Reacts with Human. Jurkat, DU 145 and HEK293T whole cell lysates were used as positive controls.
Anti-DHFR: Suitable for WB, IP, IF, IHC-P, ELISA. Reacts with Human, Mouse, Rat. HeLa and Jurkat whole cell lysates were used as positive controls.
Anti-TYMS: Suitable for WB, IHC-P, IF/ICC, Flow Cyt. Reacts with Human, Mouse, Rat, Monkey. 5-FU and lapatinib were used as positive controls.
Anti-MTHFD1: Suitable for IF, IHC-P, WB. Reacts with Human. Predicted reactivity with Mouse and Rat.
Anti-MTHFD1L: Suitable for WB, IHC-P. Reacts with Human. Colo320, HepG2 and HeLa whole cell lysates were used as positive controls.
Anti-SHMT1: Suitable for WB, IHC-P, Flow Cyt. Reacts with Human. HeLa whole cell lysate was used as positive control.
Anti-SHMT2: Suitable for IHC-P, WB, ICC/IF. Reacts with Mouse, Rat, Human. HeLa, OVCAR3 and 22Rv1 cell lysates, mouse liver and

kidney tissue lysates, were used as positive controls.
Anti-FPGS: Suitable for WB. Reacts with Human. FPGS transfected and non-transfected HEK293T whole cell lysates were used as positive and negative controls respectively.
Anti-CD11b FITC Conjugated: Suitable for Flow Cyt. Reacts with Human.
Anti-BrdU/CldU (Bio-Rad / AbD Serotec): Suitable for IF, IHC-P, Flow Cyt. Reacts with Human, Mouse, Zebrafish.
Anti-BrdU/IdU (BD Biosciences): Suitable for IF/ICC, IHC-P, Flow Cyt. Reacts with Human, Mouse, Rat, Drosophila melanogaster.

Eukaryotic cell lines

Policy information about cell lines

Cell line source(s): The following cell lines (U-2 OS, HL-60, THP-1, MV4-11, CCRF-CEM, Jurkat, SW620, CCD 841, and MCF10A) were acquired from ATCC. The cell line PL-21 was acquired from DSMZ. The lymphoblastoid cell lines LCL-534 and LCL-889 were established by Dr. Torkild Visnes in our lab. The U-2 OS cell line stably transfected with FUCCI sensor system was established by Dr. Mikael Altun in our lab. The Ewing sarcoma cell line TC71 was a gift from Dr. Oscar Fernández-Capetillo, originally established by Dr. Enrique de Alava (IBiS, Spain).

Authentication: None of the cell lines have been authenticated.

Mycoplasma contamination: Cell lines were routinely tested for mycoplasma contamination using the MycoAlert™ Mycoplasma Detection Kit (Lonza). All cell cultures used in this study tested negative to Mycoplasma contamination.

Commonly misidentified lines (See ICLAC register): None of the cell lines used in this study are listed in the ICLAC register of commonly misidentified cell lines.

Animals and other organisms

Policy information about studies involving animals; ARRIVE guidelines recommended for reporting animal research

Laboratory animals: NOD.Cg-Prkdcscid Il2rgtm1Sug/JicTac (NOG) (Taconic Biosciences, RRID: IMSR_TAC:NOG)
NOD.CB17-Prkdcscid/NCrCrl (NOD-SCID) (Charles River Laboratories, RRID: IMSR_CRL:394)
NOD.Cg-Prkdcscid Il2rgtm1Wjl/SzJ (NSG) (The Jackson Laboratory, RRID: IMSR_JAX:005557)

Approximately 6-8 weeks old female mice were used in all our efficacy studies. Mice were housed 4 per cage in individually ventilated cages (type IVC) in 12/12 light/dark cycle. Room temperature/ambient were kept at 21˚C ± 4˚C and humidity 40-70%. Animals were fed folic acid deficient diet (ENVIGO, Teklad custom diet #TD.01013) or rodent maintenance pelleted diet (SDS # 801151). Food and water were provided ad libitum.

Wild animals: No wild animals were used in this study

Field-collected samples: No field-collected samples were used in this study

Ethics oversight: All experiments in this study were performed in accordance with the guidelines from the Swedish National Board for Laboratory Animals and the European Community Council Directive (86/609/EEC) and approved by the Swedish Ethical Committee (ethical permits N217/15 and N89/14).

Note that full information on the approval of the study protocol must also be provided in the manuscript.

Flow Cytometry

Plots

Confirm that:

- [x] The axis labels state the marker and fluorochrome used (e.g. CD4-FITC).
- [x] The axis scales are clearly visible. Include numbers along axes only for bottom left plot of group (a 'group' is an analysis of identical markers).
- [x] All plots are contour plots with outliers or pseudocolor plots.
- [x] A numerical value for number of cells or percentage (with statistics) is provided.

Methodology

Sample preparation: Cell cycle analysis: HL-60 or THP-1 cells were collected, washed and re-suspended in 300 µL PBS, then fixed by adding 700 µL ice-cold absolute ethanol drop-wise while vortexing. Samples were incubated on ice for 30 min then kept at 4 °C at least overnight. Following fixation, cells were washed twice with 2% bovine serum albumin (BSA) in PBS and re-suspended in 500 µL of propidium iodide (PI) staining solution: 40 µg/mL PI, 100 µg/mL RNase A, 0.1% TritonX-100 in PBS. Cells were incubated at room temperature for 20 min then analyzed for DNA content using a Navios flow cytometer (Beckman Coulter). At least 10,000 events were acquired per sample.

Annexin V apoptosis assay: Following treatment with test compounds, HL-60 or THP-1 cells were collected together with initial culture medium, centrifuged and washed with ice-cold PBS. Per sample, 250,000 – 500,000 cells were collected and re-suspended in 100 µL 1x Binding Buffer (10 mM HEPES-NaOH pH 7.4, 140 mM NaCl, 2.5 mM $CaCl_2$), then stained at room

temperature for 15 min using the FITC-Annexin V Apoptosis Detection Kit (BD Biosciences). Following incubation with FITC-Annexin V and propidium iodide (PI), 400 µL of 1x Binding Buffer were added to the samples and immediately analyzed by flow cytometry using a Navios flow cytometer (Beckman Coulter). At least 20,000 events were acquired per sample.

Instrument	Beckman Coulter Navios Flow Cytometer, 10 colors/3 lasers (serial number AS08044)
Software	Data acquisition was performed using the Navios Software v1.5. Analysis of flow cytometry data was carried out using Kaluza Software v1.3 (Beckman Coulter)
Cell population abundance	A minimum of 10,000 events were acquired per sample following debris and doublet exclusion. Positive populations were determined using single-stain negative (unstained) and positive controls (ex. etoposide treatment for DNA damage, ATRA treatment for differentiation).
Gating strategy	Initial cell populations were gated using FSC and SSC plot of cell only (unstained) control sample to remove cell debris (small FSC vs. SSC), except in protocols where apoptotic populations were measured. Doublets and cell aggregates were excluded by gating in single cells (FSC vs. TOF). The cell population gated in after debris and doublet exclusion was then used to create single-staining histograms (cell cycle based on PI, cell differentiation based on anti-CD11b-FITC antibody, DNA damage based on anti-gamma-H2AX antibody) and double-staining quadrants (PI and anti-annexin V-FITC for apoptosis assays, PI and CD11b-FITC for differentiation assays, Hoechst and EdU detected by azide click chemistry for cell cycle analysis).

☒ Tick this box to confirm that a figure exemplifying the gating strategy is provided in the Supplementary Information.
