# Supplementary figures and images for: Pharmacological targeting of MTHFD2 suppresses acute myeloid leukemia by inducing thymidine depletion and replication stress
Source: Nat Cancer. 2022 Feb 28;3(2):156–72. doi: 10.1038/s43018-022-00331-y (PMC8885417; doi:10.1038/s43018-022-00331-y)

**Source Data - Unprocessed images of Western blots related to Figure 2d.**

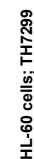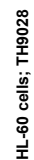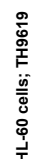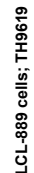

Supplement: Source Data Fig. 2 — Unprocessed western blots. [file 43018_2022_331_MOESM6_ESM.pdf]

Source Data - Unprocessed images of Western blots related to Figure 6d.

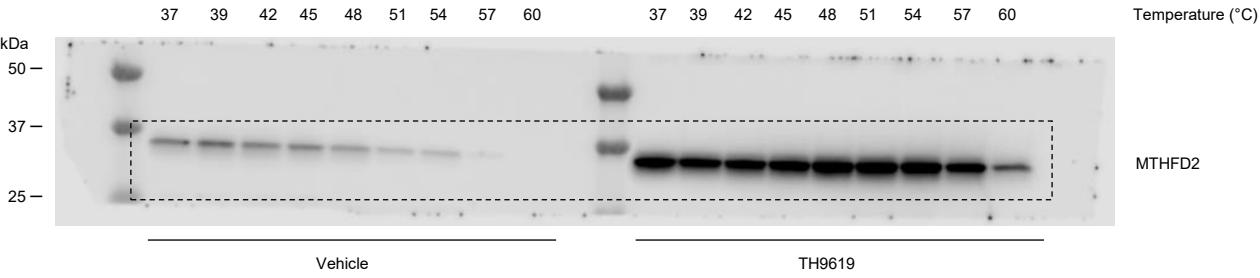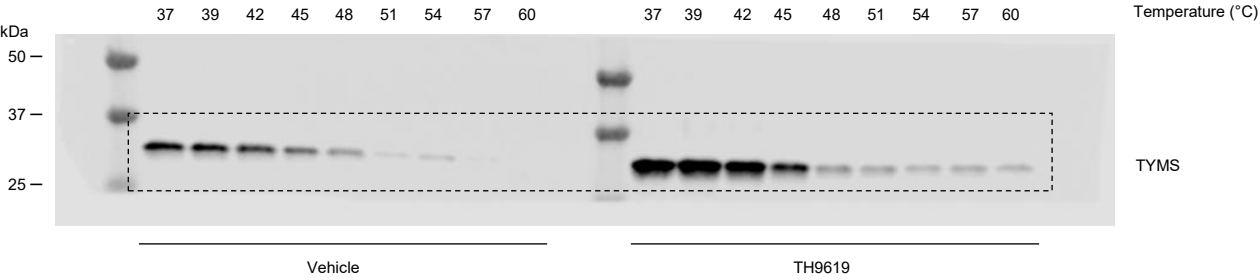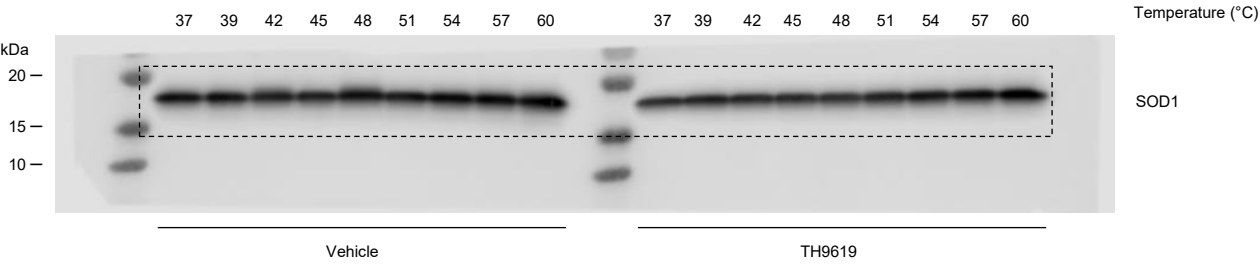

Supplement: Source Data Fig. 6 — Unprocessed western blots. [file 43018_2022_331_MOESM12_ESM.pdf]

**Source Data - Unprocessed images of Western blots related to Extended Data Figure 4b.**

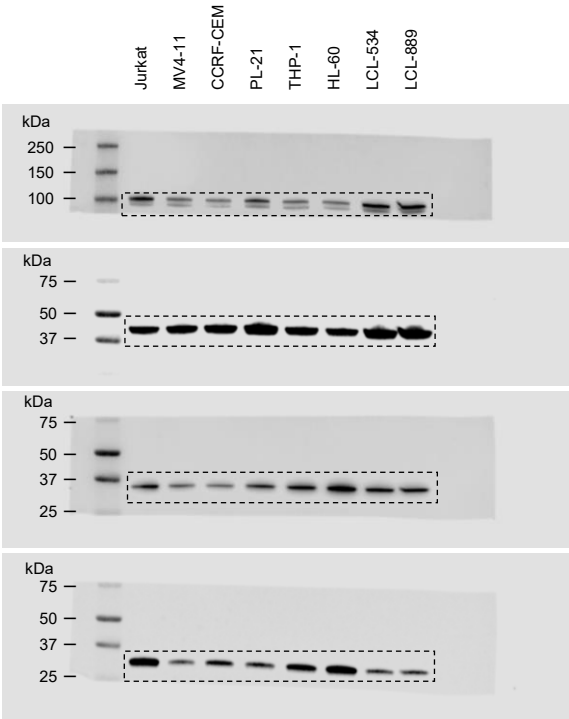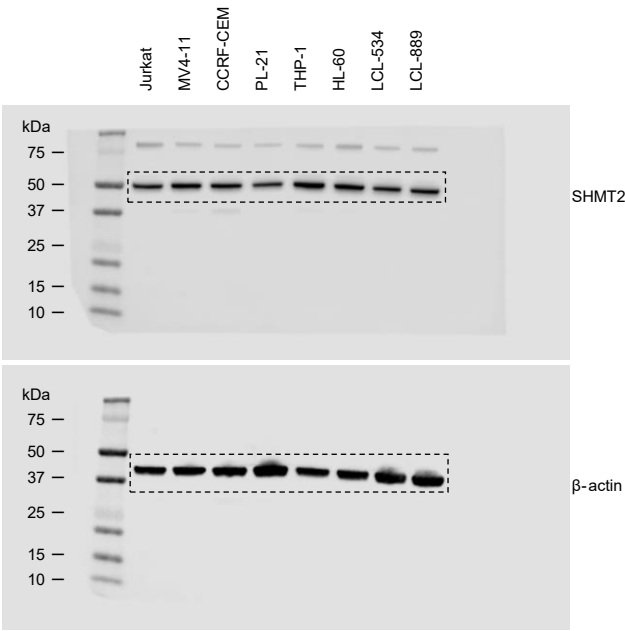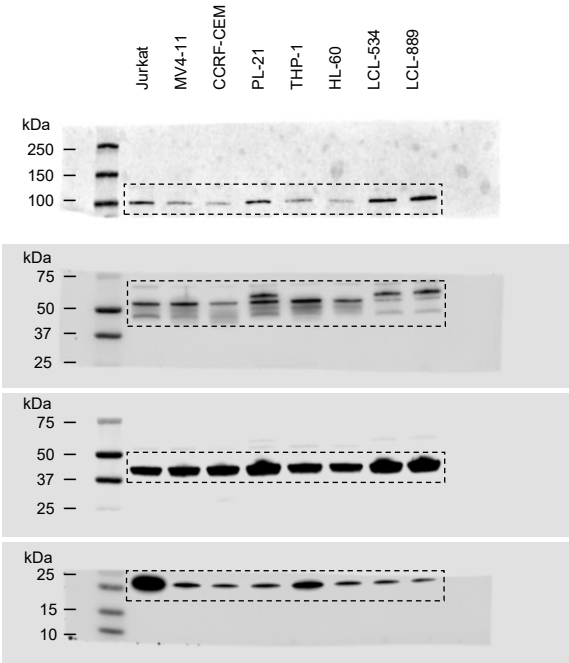

Supplement: Source Data Extended Data Fig. 4 — Unprocessed western blots. [file 43018_2022_331_MOESM17_ESM.pdf]

Source Data - Uncropped images of Western blots related to Extended Data Figure 10e.

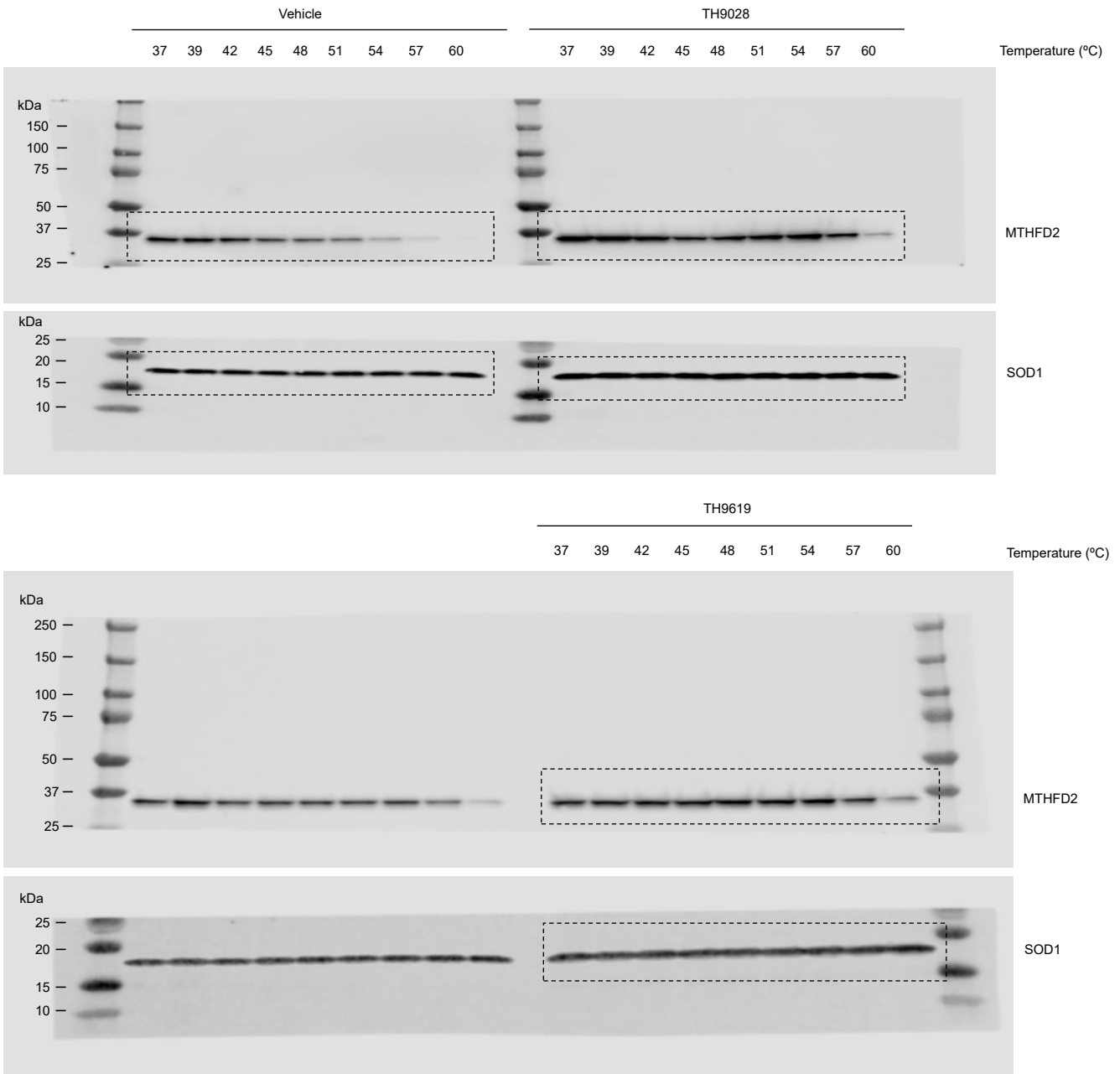

Supplement: Source Data Extended Data Fig. 10 — Unprocessed western blots. [file 43018_2022_331_MOESM24_ESM.pdf]
