# Supplementary material for: Pharmacological targeting of MTHFD2 suppresses acute myeloid leukemia by inducing thymidine depletion and replication stress
Source: Nat Cancer. 2022 Feb 28;3(2):156–72. doi: 10.1038/s43018-022-00331-y (PMC8885417; doi:10.1038/s43018-022-00331-y)

Source Data - Unprocessed images of Western blots related to Figure 4d.

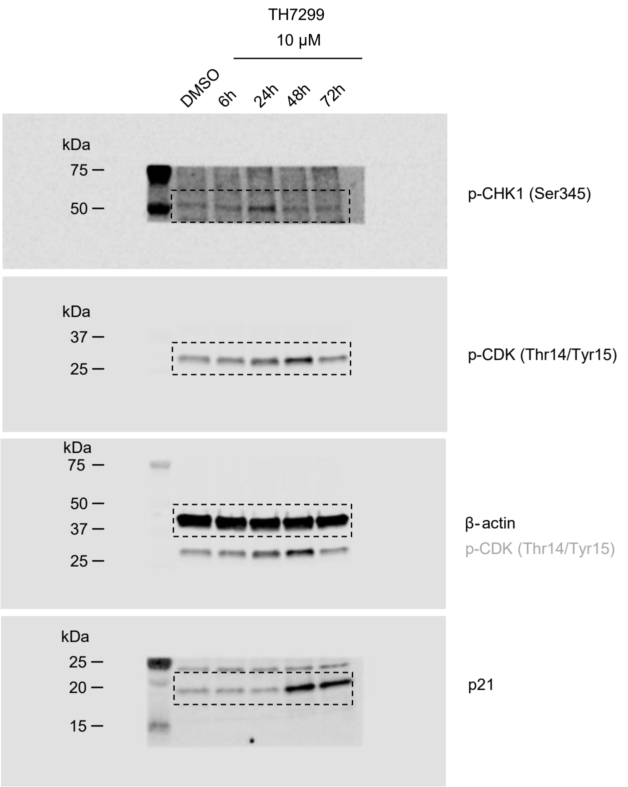

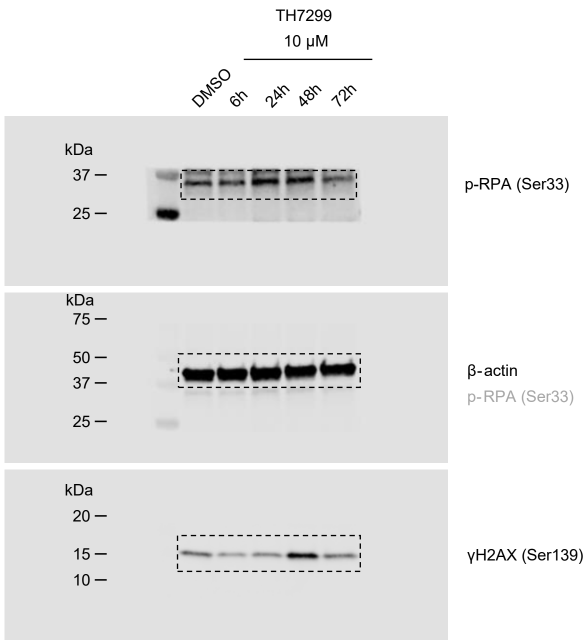

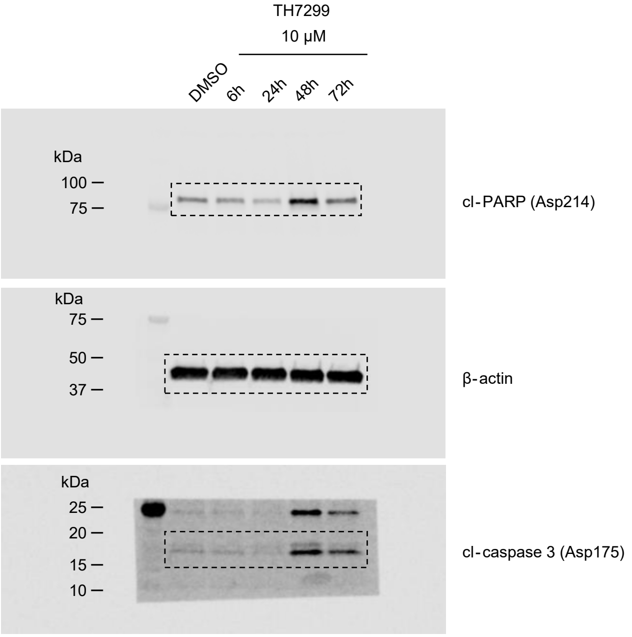

Source Data - Unprocessed images of Western blots. Blots related to Figure 4e.

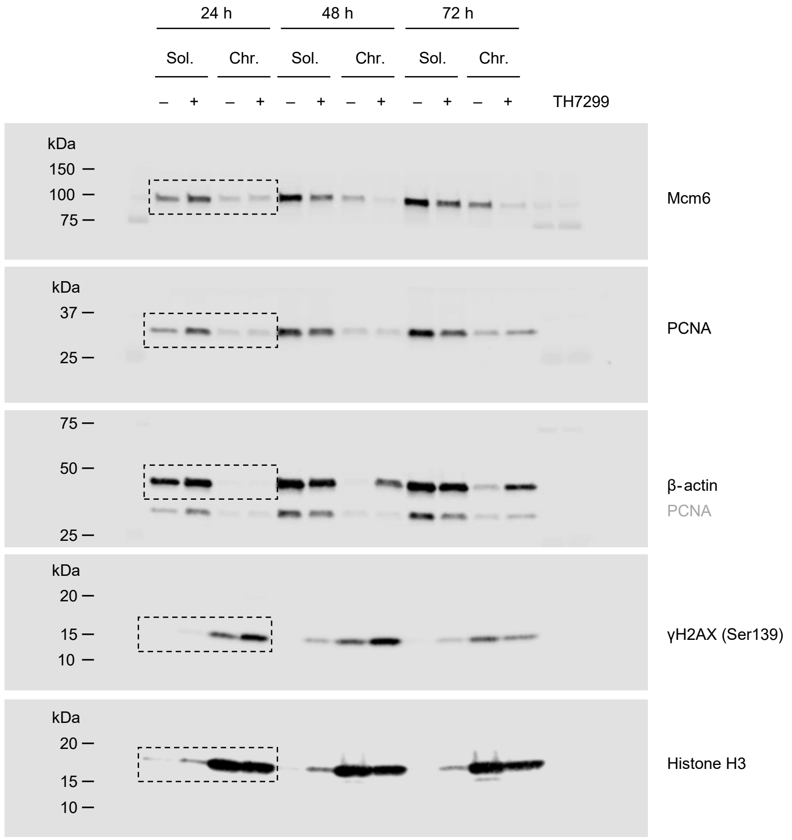

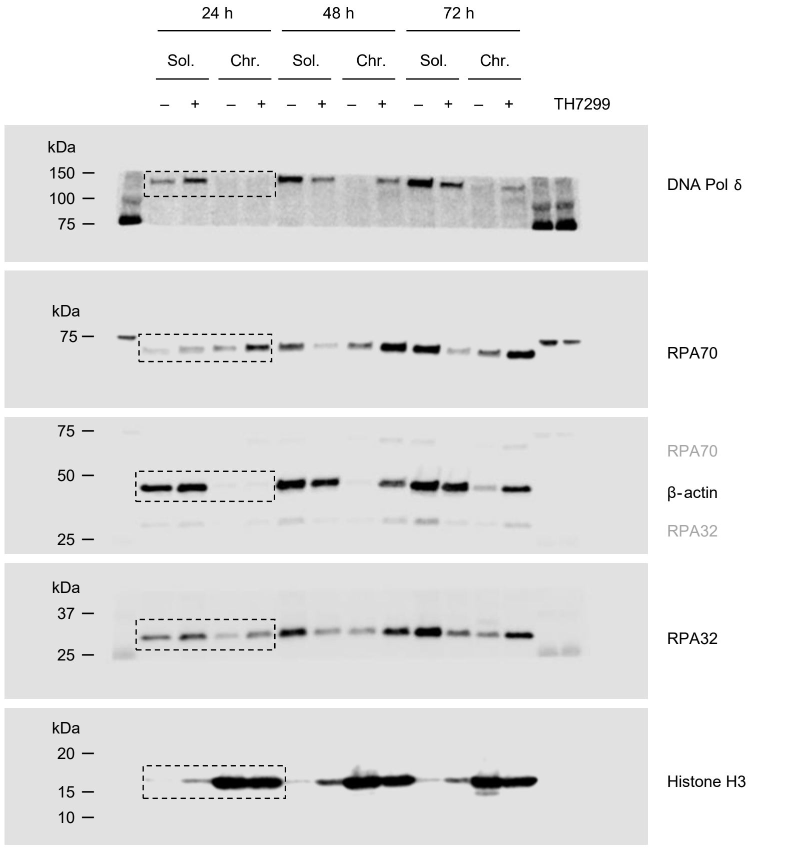

Supplement: Source Data Fig. 4 — Unprocessed western blots. [file 43018_2022_331_MOESM9_ESM.pdf]
