# Supplementary material for: Pharmacological targeting of MTHFD2 suppresses acute myeloid leukemia by inducing thymidine depletion and replication stress
Source: Nat Cancer. 2022 Feb 28;3(2):156–72. doi: 10.1038/s43018-022-00331-y (PMC8885417; doi:10.1038/s43018-022-00331-y)

Source Data - Unprocessed images of Western blots related to Extended Data Figure 2h.

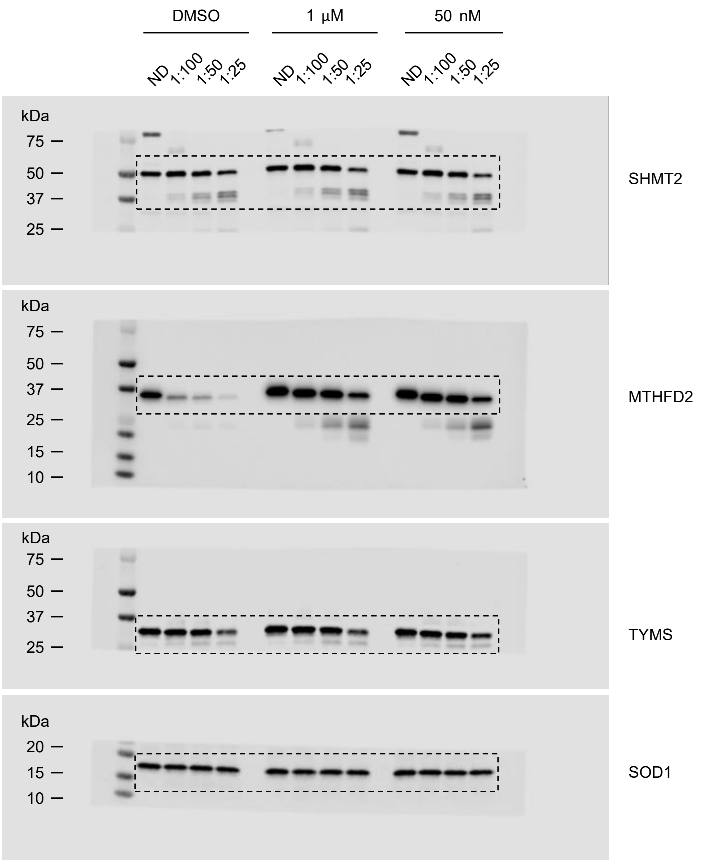

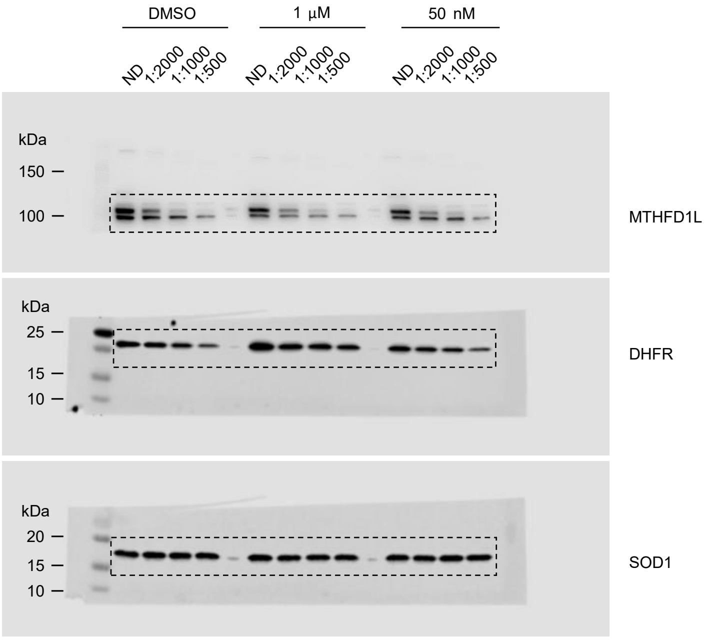

Source Data - Unprocessed images of Western blots related to Extended Data Figure 2i.

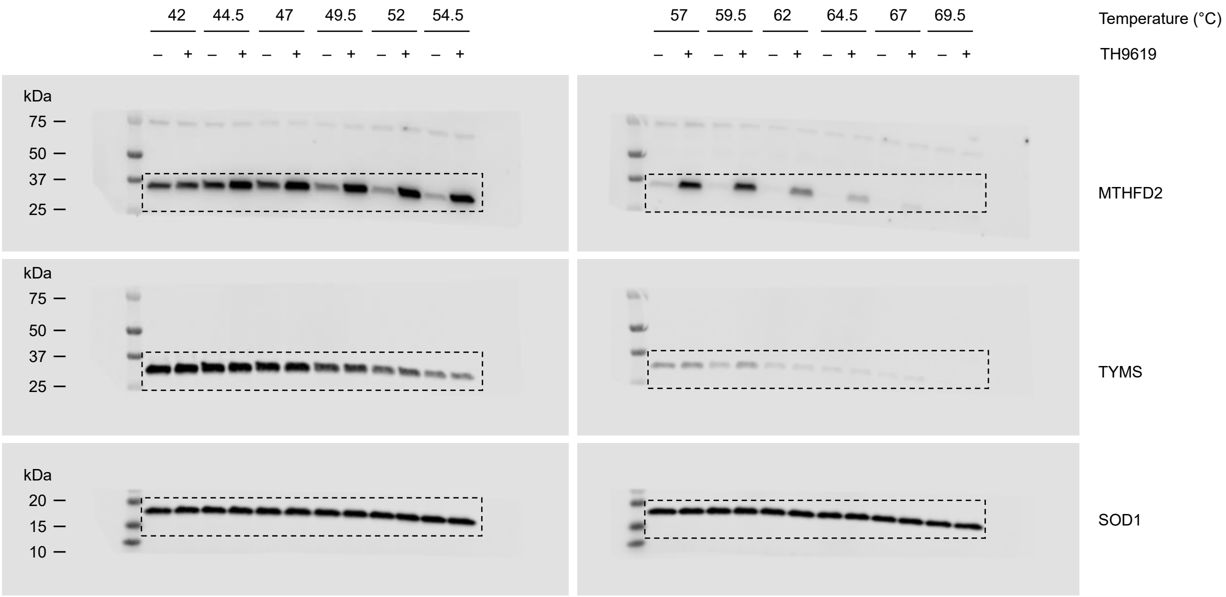

Source Data - Unprocessed images of Western blots related to Extended Data Figure 2l.

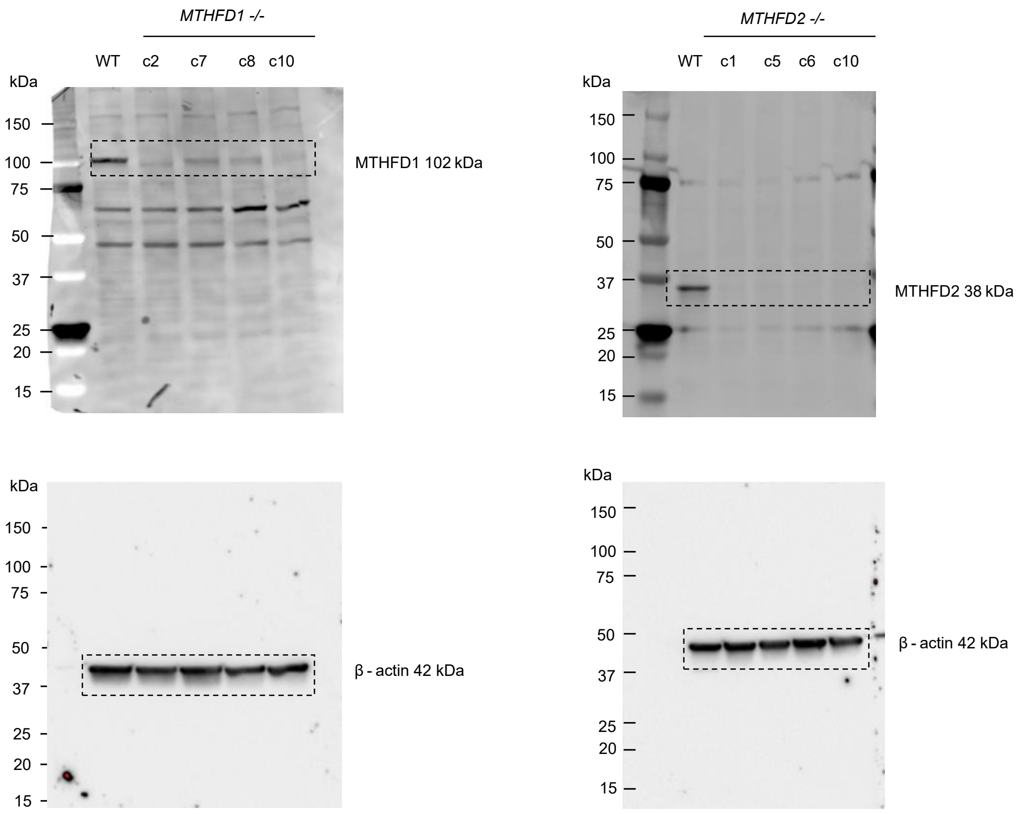

Supplement: Source Data Extended Data Fig. 2 — Unprocessed western blots. [file 43018_2022_331_MOESM15_ESM.pdf]
